# Minimal invasive extracorporeal circulation versus conventional cardiopulmonary bypass in cardiac surgery: a contemporary systematic review and meta-analysis

**DOI:** 10.1093/ejcts/ezaf112

**Published:** 2025-03-25

**Authors:** Kyriakos Anastasiadis, Polychronis Antonitsis, Christos Voucharas, Fani Apostolidou-Kiouti, Apostolos Deliopoulos, Anna-Bettina Haidich, Helena Argiriadou

**Affiliations:** Cardiothoracic Department, Aristotle University of Thessaloniki School of Medicine, Thessaloniki, Greece; Cardiothoracic Department, Aristotle University of Thessaloniki School of Medicine, Thessaloniki, Greece; Cardiothoracic Department, Aristotle University of Thessaloniki School of Medicine, Thessaloniki, Greece; Department of Hygiene, Social-Preventive Medicine and Medical Statistics, Aristotle University of Thessaloniki School of Medicine, Thessaloniki, Greece; Cardiothoracic Department, Aristotle University of Thessaloniki School of Medicine, Thessaloniki, Greece; Department of Hygiene, Social-Preventive Medicine and Medical Statistics, Aristotle University of Thessaloniki School of Medicine, Thessaloniki, Greece; Department of Anesthesiology and Intensive Care, Aristotle University of Thessaloniki School of Medicine, Thessaloniki, Greece

**Keywords:** Minimal invasive extracorporeal circulation, Cardiopulmonary bypass, Extracorporeal circulation, Coronary artery bypass grafting, Meta-analysis

## Abstract

**OBJECTIVES:**

The question whether minimally invasive extracorporeal circulation (MiECC) represents the optimal perfusion strategy in cardiac surgery remains unanswered. We sought to systematically review the entire literature and thoroughly address the impact of MiECC versus conventional cardiopulmonary bypass (cCPB) on adverse clinical outcomes after cardiac surgery.

**METHODS:**

We searched PubMed, Scopus and Cochrane databases for appropriate articles as well as conference proceedings from major congresses up to 31 August 2024. All randomized controlled trials (RCTs) that fulfilled pre-defined MiECC criteria were included in the analysis. The primary outcome was mortality, while morbidity and transfusion requirements were secondary outcomes. The risk of bias was assessed using the Cochrane Risk of Bias 2 tool. All studies meeting the outcomes of interest of this systematic review were eligible for synthesis.

**RESULTS:**

Of the 738 records identified, 36 RCTs were included in the meta-analysis with a total of 4849 patients. MiECC was associated with significantly reduced mortality [odds ratio (OR) 0.66; 95% confidence interval (CI) 0.53–0.81; *P* = 0.0002; *I*^2^ = 0%] as well as risk of postoperative myocardial infarction (OR 0.42; 95% CI 0.26–0.68; *P* = 0.002; *I*^2^ = 0%) and cerebrovascular events (OR 0.55; 95% CI 0.37–0.80; *P* = 0.007; *I*^2^ = 0%). Moreover, MiECC reduced RBC transfusion requirements, blood loss and rate of re-exploration for bleeding together with incidence of atrial fibrillation. This resulted in significantly reduced duration of mechanical ventilation, ICU and hospital stay.

**CONCLUSIONS:**

This meta-analysis provides robust evidence for the beneficial effect of MiECC in reducing postoperative morbidity and mortality after cardiac surgery and prompts for a wider adoption of this technology.

## GRAPHICAL ABSTRACT

**Figure:**
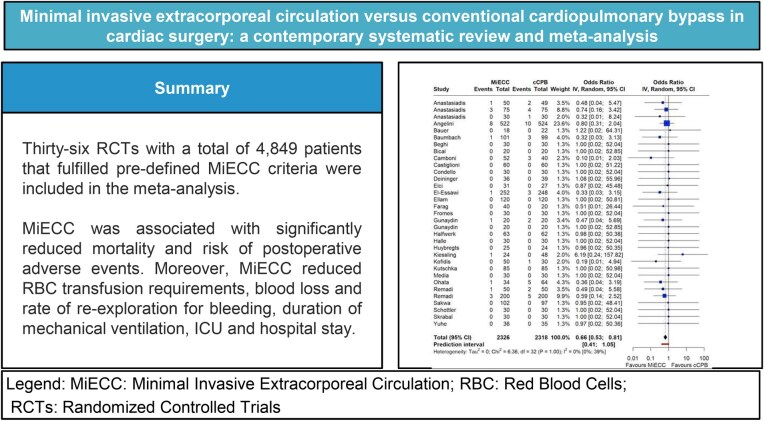

## INTRODUCTION

Despite the considerable improvements in cardiac surgical techniques, the incidence of postoperative morbidity and mortality after cardiac surgery remains substantial, as consistently demonstrated by large-scale registry data [1]. A major contributing factor to these adverse outcomes is the inherent non-physiological nature of cardiopulmonary bypass (CPB), which introduces significant alterations in normal circulatory dynamics [2]. The advent of minimally invasive extracorporeal circulation (MiECC) aimed to mitigate the invasive nature of conventional CPB [3]. This technology is not limited just to an advanced perfusion circuit, but it represents a strategic shift towards a multidisciplinary strategy for a more ‘physiologic’ perfusion [4]. It integrates all contemporary advancements in the field of circuit design and perfusion technology in one system complemented by surgical, anesthesiological and perfusion techniques (i.e. goal-directed perfusion, point-of-care coagulation monitoring).

Our group published, more than a decade ago, a seminal meta-analysis which showed, for the first time, a mortality benefit favouring mini-CPB systems [5]. Since then, several randomized controlled trials (RCTs) and meta-analyses have highlighted the benefit of miniaturized CPB over conventional set-up [6, 7]. Despite the consistently shown perceived advantages of MiECC in improving clinical outcomes, the joint 2019 European Association for Cardio-Thoracic Surgery (EACTS), European Association of Cardiothoracic Anaesthesiology and Intensive Care (EACTAIC) and the European Board of Cardiovascular Perfusion guidelines on CPB assigned a class IIa level of evidence B recommendation for the use of MiECC in cardiac surgery [2]. The major criticism lies in study design as well as in the heterogeneity of mini-CPB systems used, many of which did not fully comply with MiECC criteria as defined in Minimal Invasive Extracorporeal Technologies International Society (MiECTiS) consensus published in 2016 [4].

The recent publication of the COMICS trial results provides a valuable addition to the existing body of evidence [8]. Despite the unforeseen impact of the COVID-19 pandemic, which led to the premature cessation of the trial, COMICS demonstrated a significant reduction in the risk of serious adverse events, as captured by a composite primary outcome, alongside a significant improvement in patient-reported quality of life. In the light of the new evidence, a well-designed meta-analysis comparing MiECC with any other available perfusion circuit is considered useful so as to clarify the benefit from this technology. Therefore, we set out to systematically review the entire literature and thoroughly address the impact of MiECC versus conventional CPB (cCPB) on adverse clinical outcomes after cardiac surgery.

## PATIENTS AND METHODS

This analysis was prospectively registered on the International Prospective Register of Systematic Reviews in Health and Social Care (PROSPERO, ID: CRD42024555217). Ethical and IRB approval were not required because no human or animal subjects were involved. The present study was conducted according to the Preferred Reporting Items for Systematic Reviews and Meta-Analyses (PRISMA) guidelines [9].

### Search strategy

The full search strategy is presented in Supplementary Material, Table S1. Two independent investigators (C.V. and F.A.-K.) searched PubMed, Scopus and Cochrane databases for appropriate articles up to 31 August 2024. The search strategy aimed to include all the RCTs performed and published on the topic. The reference lists of the retrieved articles and reviews on the topic were checked for further potentially relevant publications (backward snowballing). Moreover, hand or computerized search involving conference proceedings from MiECTiS, EACTS, Society of Thoracic Surgeons and European Society for Cardiovascular Surgery congresses was performed (2004–2024).

### Selection criteria

All titles and abstracts were reviewed against pre-defined inclusion and exclusion criteria. Studies were considered for inclusion if they were written in English and reported direct comparison between adult patients who underwent cardiac surgery with MiECC versus cCPB. In order to exclude bias from small underpowered studies, only RCTs with a minimum of 40 patients in both groups were included in the analysis. Moreover, only studies that fulfilled the universally accepted MiECC criteria, as defined in the 2016 MiECTiS position paper, were considered eligible for inclusion in the meta-analysis [4]. All other mini-bypass configurations and MiECC-like circuit designs were excluded from the analysis so as to reduce the risk of heterogeneity within the MiECC group. Thus, in the present meta-analysis MiECC comprised a closed biocompatible low-prime volume circuit, baring a centrifugal pump, with absence of venous or cardiotomy reservoir precluding blood-air contact. Shed-mediastinal blood was retrieved exclusively by a cell-saving device. Venting lines, when incorporated, were driven into the cell-saver or into a vacuum bag reservoir. On the other hand, any system comprising an open venous reservoir with cardiotomy suction that collects shed blood and returns it into the circuit was considered as cCPB. Other exclusion criteria included lack of outcome data and duplicate publication (in which case the most recent article or the one with the largest cohort of patients was selected).

### Data extraction

The full text was assessed for eligibility by 2 reviews who extracted quantitative data (P.A., C.V.). Conflicts in extracted data were resolved by a third and fourth reviewers (F.A.-K., K.A.). The following data were extracted from each of the included studies into a pre-defined database: type of MiECC circuit, sample size, surgical procedure and CPB characteristics. The pre-specified primary outcome was mortality assessed as per the protocol and definition of the individual study. The pre-specified secondary outcomes were: postoperative myocardial infarction (as defined in individual study); cerebrovascular events (stroke and transient ischaemic attacks); acute renal failure (as defined in the individual study); need of red blood cell (RBC) or fresh frozen plasma (FFP) transfusion; haemodilution (defined as haematocrit drop after CPB); preservation of platelet count; postoperative blood loss and rate of re-exploration for bleeding; new onset of atrial fibrillation; myocardial protection [defined as peak troponin and creatine kinase (CK) release, need for inotropic support, incidence of low cardiac output syndrome and intra-aortic balloon pump use postoperatively]; time on mechanical ventilation, intensive care unit (ICU) stay and time to hospital discharge. Supplementary Material, Table S2 presents definitions of the outcomes for each study.

### Risk of bias assessment

The risk of bias for RCTs was assessed using the Cochrane risk of bias 2 tool (RoB2) developed according to the Cochrane Collaboration guidelines for RCTs, which evaluates the risk of bias in 5 areas (randomization process, deviation from the intended intervention, missing data, measurement the outcome and selection for the reported result) [10]. For each trial, 2 authors (C.V. and F.A.-K.) independently assessed the risk of bias associated with each area. Any conflict on the attribution of the risk of bias was resolved by a consensus.

### Statistical analysis

All studies meeting the inclusion criteria and reporting the outcomes of interest, even as secondary outcomes, were eligible for synthesis. There were no transformations for the primary outcome. The number of transfused blood products was converted to transfusion rate when there was available data. The remaining outcomes did not require any data transformation. The results of the synthesis were presented using forest plots.

A random effects meta-analytic model was used for dichotomous and continuous outcomes due to the expected heterogeneity. Restricted maximum likelihood was used to estimate the heterogeneity variance which was quantified with the τ^2^ to calculate the *I*^2^ statistic. In a single, secondary outcome (intra-aortic balloon pump) maximum likelihood was used to estimate heterogeneity due to algorithm non-convergence. Overall effect was expressed as odds ratio (OR) for dichotomous outcomes and mean difference for continuous outcomes. A single instance where standardized mean difference had to be used was encountered (FFP transfusion). The 95% confidence intervals (CIs) were computed under the Hartung–Knapp–Sidik–Jonkman modification. Prediction intervals accompany the CIs and were calculated using the Higgins–Thompson–Spiegelhalter method due to the large number of included studies. Multivariate meta-analysis was *a priori* designed to combine multiple binary outcomes into a composite outcome but the lack of individual patient data and the rareness of the events that would comprise the composite outcome did not allow for such an approach. To account for the number of zero studies included and the classification of the primary outcome as a rare event, additional models were implemented: the Mantel–Haenszel model for risk difference, a generalized linear mixed-effects model with random intercept and a rare event meta-analysis model as proposed by Zabriskie *et al.* [11]. This is the first update of a previous meta-analysis, consequently no method to assess the increase of type I error was implemented. Publication bias was assessed both by visually inspecting funnel plots and by performing Egger’s test when the number of included studies was >10.

Only 1 subgroup analysis was planned, and it focused on the type of surgery performed. The meta-analysis was divided into 2 groups: coronary artery bypass grafting (CABG)—only and all other types of surgery. Data were extracted from published reports, Supplementary Material and requests from authors. Sensitivity analyses were conducted to assess the impact of risk of bias differences across domains. The metafor, mvmeta, metavcov and rema packages for R v4.3.3 were used to run all analyses.

## RESULTS

### Study selection

Database searching yielded a total of 738 studies, whereas 21 more records were retrieved from congresses proceedings. After exclusion of 520 inappropriate records, 218 were screened and 77 were retrieved as complete articles and assessed for eligibility according to the pre-specified inclusion criteria. After removing 7 duplicates, 10 non-randomized and 21 RCTs and abstracts that did not fulfil the inclusion criteria, 39 RCTs were considered for inclusion in the final analysis. After scrutiny evaluation, 3 RCTs were found not fulfilling pre-defined MiECC criteria [12–14]. Thus, 36 RCTs were included in the meta-analysis with a total of 4849 patients. The PRISMA flow diagram is presented in Supplementary Material, Fig. S1.

### Study characteristics

Tables 1 and 2 summarize patient as well as CPB circuit characteristics and heparin management. Of the total 4849 patients, 2429 were allocated to MiECC, whereas 2420 were allocated to cCPB. The majority of patients (3797/4849; 78.3%) were operated for CABG (1902 operated on MiECC vs 1895 operated on cCPB), while 776 patients (16%) underwent aortic valve replacement (AVR) (388 operated on MiECC vs 388 operated on cCPB). The remaining 276 patients (5.7%) had complex or mitral surgery. The most common system used was the Maquet MECC system, while warm-blood cardioplegia was primarily administered. As expected from the difference in circuit design, priming volume was significantly reduced in MiECC compared to cCPB (637 ± 204 vs 1521 ± 235 ml; *P* < 0.001). Regarding procedural characteristics, MiECC was associated with reduced CPB time [weighted mean difference (WMD) = −2.02 (−5.17,1.13); *P* < 0.001; *I*^2^ = 79%)] (Supplementary Material, Fig. S2) and similar cross-clamp time [WMD = 0.21 (−1.61, 2.02); *P* = 0.8; *I*^2^ = 64%)] (Supplementary Material, Fig. S3).

**Table 1:** Authors, journals, year of publication, surgical procedure and number of included patients

Author	Journal	Year	Procedure	Patients	MiECC	cCPB
Fromes *et al.* [15]	*Eur J Cardiothor Surg*	2002	CABG	60	30	30
Remadi *et al.* [16]	*J Thorac Cardiovasc Surg*	2004	AVR	100	50	50
Beghi *et al.* [17]	*Ann Thorac Surg*	2006	CABG	60	30	30
Bical *et al.* [18]	*Eur J Cardiothor Surg*	2006	AVR	40	20	20
Remadi *et al.* [19]	*Am Heart J*	2006	CABG	400	200	200
Huybregts *et al.* [20]	*Ann Thorac Surg*	2007	CABG	49	25	24
Skrabal *et al.* [21]	*ASAIO J*	2007	CABG	60	30	30
Valtonen *et al.* [22]	*Scand Cardiovasc J*	2007	CABG	40	20	20
Perthel *et al.* [23]	*Eur J Cardiothor Surg*	2007	CABG	60	30	30
Kofidis *et al.* [24]	*Perfusion*	2008	CABG	80	50	30
Ohata *et al.* [25]	*ASAIO J*	2008	CABG	98	34	64
Schöttler *et al.* [26]	*Thorac Cardiovasc Surg*	2008	CABG	60	30	30
Castiglioni *et al.* [27]	*Interact Cardiovasc Thorac Surg*	2009	AVR	120	60	60
Kutschka *et al.* [28]	*Perfusion*	2009	CABG ± AVR/aortic root surgery	170	85	85
Gunaydin *et al.* [29]	*Perfusion*	2009	CABG	40	20	20
Sakwa *et al.* [30]	*J Thorac Cardiovasc Surg*	2009	CABG	199	102	97
Camboni *et al.* [31]	*ASAIO J*	2009	CABG	92	50	40
Anastasiadis *et al.* [32]	*Perfusion*	2010	CABG	99	50	49
Bauer *et al.* [33]	*J Extra Corpol Technol*	2010	CABG	40	18	22
El-Essawi *et al.* [34]	*Perfusion*	2011	CABG, AVR, CABG+AVR	500	252	248
Abdel Aal *et al.* [35]	*Interact CardioVasc Thorac Surg*	2011	CABG	80	40	40
Anastasiadis *et al.* [36]	*Eur J Cardiothor Surg*	2016	CABG	60	30	30
Baumbach *et al.* [37]	*Ann Thorac Surg*	2016	mini AVR, MVR	200	101	99
Deininger *et al.* [38]	*Thorac Cardiovasc Surg*	2016	CABG	75	36	39
Anastasiadis *et al.* [39]	*Artif Organs*	2017	CABG	150	75	75
Farag *et al.* [40]	*Artif Organs*	2017	CABG	60	40	20
Kiessling *et al.* [41]	*Heart Surg Forum*	2018	CABG	72	24	48
Elci *et al.* [42]	*Cardiol Res Pract*	2019	CABG	58	31	27
Halfwerk *et al.* [43]	*Ann Thorac Surg*	2019	AVR	125	63	62
Yuhe *et al.* [44]	*Ann Card Anaesth*	2020	CABG	71	36	35
Media *et al.* [45]	*Perfusion*	2021	CABG	60	30	30
Condello *et al.* [46]	*Interact CardioVasc Thorac Surg*	2021	CABG	60	30	30
Gunaydin *et al.* [47]	*Perfusion* (abstract)	2021	CABG	40	20	20
Ellam *et al.* [48]	*Perfusion*	2023	CABG	240	120	120
Angelini *et al.* [8]	*Perfusion*	2024	CABG, AVR, CABG+AVR	1071	535	536
Halle *et al.* [49]	*J Cardiothorac Surg*	2024	CABG	60	30	30

AVR: aortic valve replacement; CABG: coronary artery bypass grafting; cCPB: conventional cardiopulmonary bypass; MiECC: minimal invasive extracorporeal circulation.

**Table 2:** Cardiopulmonary bypass circuit characteristics of included studies

Author	MiECC manufacturer	Type MiECC	Priming MiECC (ml)	Priming cCPB (ml)	cCPB characteristics	Cardioplegia	ACT MiECC	ACT cCPB	Heparin MiECC (IU/kg)	Heparin cCPB (IU/kg)
Fromes *et al.* [15]	Maquet MECC	I	500	NR	HC/CS	WB	NR	NR	300	300
Remadi *et al.* [16]	Maquet MECC	I	450	1700	NC/CS	WB	400	400	150	300
Beghi *et al.* [17]	Maquet MECC	I	450	1500	NC	WB	NR	NR	150	300
Bical *et al.* [18]	Maquet MECC	I	630	1760	SMAC	WB	NR	NR	300	300
Remadi *et al.* [19]	Maquet MECC	I	450	1700	NC/CS	WB	400	400	150	300
Huybregts *et al.* [20]	Synergy mini-bypass (Cobe)	II	393	1330	PC/CP/SSVR	CC	480	480	400	400
Skrabal *et al.* [21]	Maquet MECC	I	500	1500	HC	WB	250	400	200–350	200–350
Valtonen *et al.* [22]	ECC.O (Dideco)	III	1100	2100	PC	CB	480	480	300	300
Perthel *et al.* [23]	ECC.O (Dideco)	II	700	1800	PC/CS	WB	480	480	300	300
Kofidis *et al.* [24]	Maquet MECC	I	NR	NR	NC	WB	NR	NR	NR	NR
Ohata *et al.* [25]	Capiox (Terumo)	III	750	1600	PMEAC/CP	CB	NR	NR	300	300
Schöttler *et al.* [26]	Maquet MECC	III	900	1700	NC	WB	NR	NR	NR	NR
Castiglioni *et al.* [27]	Maquet MECC	I	500	1600	PC/CS	CB	480	480	300	300
Kutschka *et al.* [28]	ROCsafe^TM^ MPC (Terumo)	III	800	1700	NC/CP	WB/CC	NR	NR	NR	NR
Gunaydin *et al.* [29]	ROCsafe^TM^ MPC (Terumo)	II	850	1360	NC	WB	480	480	300	300
Sakwa *et al.* [30]	Medtronic Resting Heart	II	900	1850	NC/CP	NR	400	400	350	350
Camboni *et al.* [31]	Maquet MECC; PRECiSe Medos; Medtronic Resting Heart	II	500	1200	NC	WB	NR	NR	NR	NR
Anastasiadis *et al.* [32]	Maquet MECC	II	500	1500	NC	WB	300	450	150	300
Bauer *et al.* [33]	Maquet MECC	II	872	1630	NC	WB	NR	NR	IHM	IHM
El-Essawi *et al.* [34]	ROCsafe^TM^ MPC (Terumo)	III/IV	600	1500	NC	WB/CC	480	480	300	300
Abdel Aal *et al.* [35]	Medtronic Resting Heart	II	680	1700	NC	WB	360	480	NR	300–400
Anastasiadis *et al.* [36]	Medtronic Inc.	IV	500	1500	NC	WB	300	450	150	300
Baumbach *et al.* [37]	Maquet MECC	II	225	1337	PMEAC	WB	400	400	350–400	350–400
Deininger *et al.* [38]	Maquet MECC	I	600	1250	NC	WB	400	400	350	350
Anastasiadis *et al.* [39]	Maquet MECC	I	500	1500	NC	WB	300	450	150	300
Farag *et al.* [40]	Maquet MECC/ECC.O (Dideco)	II	750	1100	NC/CS	WB	350	350	300	300
Kiessling *et al.* [41]	Maquet MECC	II	600	1290	HC	WB/CB	350	450	NR	NR
Elci *et al.* [42]	Maquet MECC	II	800	1650	NC	WB	300	400	150	300
Halfwerk *et al.* [43]	Maquet MECC	II	800	1500	HC/CP/SSVR	WB	440	440	400	400
Yuhe *et al.* [44]	Stockert ΗLM	ΙΙ	800-900	1300-1400	PC/CS	NR	NR	NR	NR	NR
Media *et al.* [45]	Medtronic Inc.	III	400	1400	NC	CB	400	400	IHM	IHM
Condello *et al.* [46]	Stockert S5 HLM	III	450	1250	PC/VAVD	WB	NR	NR	NR	NR
Gunaydin *et al.* [47]	LivaNova	IV	NR	1200	PC	NR	NR	NR	NR	NR
Ellam *et al.* [48]	Maquet MECC	III	1000	2000	HC	WB/TB	480	480	IHM	IHM
Angelini *et al.* [8]	Multiple systems	II/III/IV	750	1250	HC/CP/CS	WB/CB/CC	400	480	150/300	300
Halle *et al.* [49]	Medtronic Inc.	III	400	1400	NC	CB	400	400	IHM	IHM

ACT: aActivated clotting time; CB: cold blood; CC: cold crystalloid; cCPB: conventional cardiopulmonary bypass; CP: centrifugal pump; CS: cell salvage; HC: heparin coated; IHM: individualized heparin management; MiECC: minimal invasive extracorporeal circulation; NC: non-coated; NR: not reported; PC: phosphorylcholine coated; PMEAC: poly-2-methoxyethyl acrylate coated; SMAC: surface-modifying additives coating; SSVR: soft shell venous reservoir; TB: tepid blood; VAVD: vacuum-assisted venous drainage; WB: warm blood.

### Primary outcome

**Figure 1:**
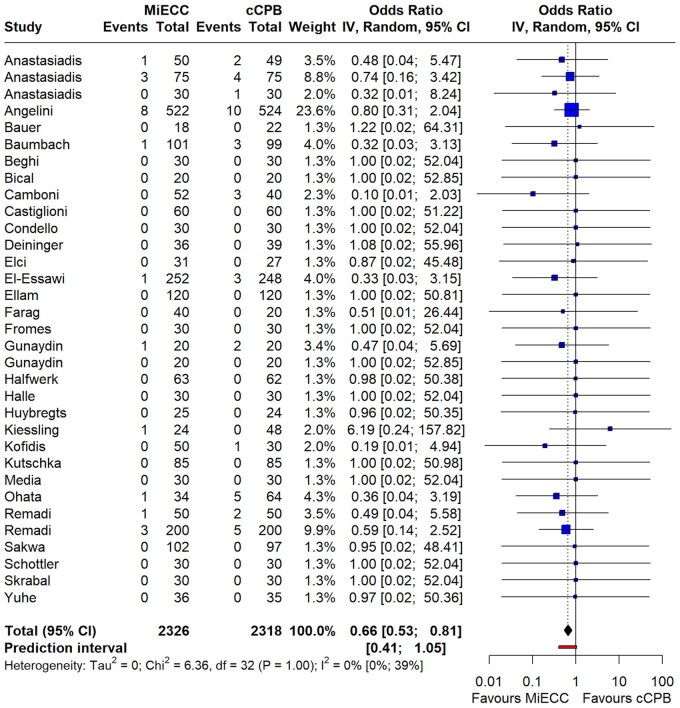
Forest plot of randomized trials comparing mortality in patients operated with minimally invasive extracorporeal circulation (MiECC) versus conventional cardiopulmonary bypass (cCPB). A significant reduction in mortality (*P* = 0.002) was observed with the use of MiECC.

There were 33 studies reporting on mortality, the majority of which were zero studies (double zero studies: 20/33, single zero studies: 4/33). A significant mortality benefit was observed with MiECC (OR 0.66; 95% CI 0.53–0.81; *P* = 0.0002; *I*^2^ = 0%) (Fig. 1). The mortality benefit of MiECC was evidenced in both coronary and non-coronary surgery subgroups, though statistical significance was detected only in the CABG subgroup (OR 0.61; 95% CI 0.48–0.79; *P* < 0.001 for CABG vs OR 0.70; 95% CI 0.29–1.67; *P* = 0.3 for non-coronary surgery) (Supplementary Material, Fig. S4). Sensitivity analyses did not alter the direction or the significance of the initial model results. The funnel plot showed no evidence of small-study effect (Supplementary Material, Fig. S5—Egger’s test for funnel plot asymmetry, *P* = 0.5029). The risk of bias, according to RoB2 tool, was attributed mainly to lack of reporting of the randomization process and of an analysis plan (Supplementary Material, Fig. S6). Sensitivity analyses excluding high-risk bias studies showed no difference in the direction or significance of the results (Supplementary Material, Fig. S7).

### Secondary outcomes

**Figure 2:**
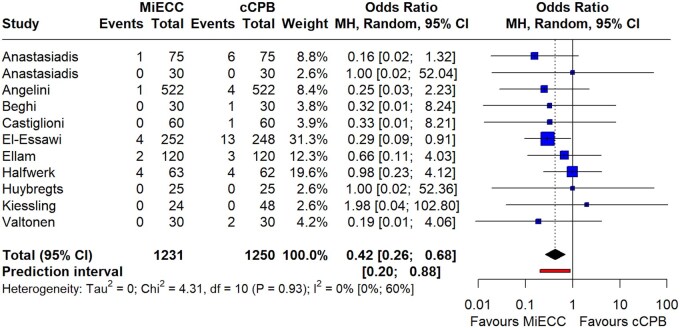
Forest plot of randomized trials comparing the incidence of postoperative myocardial infarction in patients operated with minimally invasive extracorporeal circulation (MiECC) versus conventional cardiopulmonary bypass (cCPB). A significant reduction in the rate of postoperative myocardial infarction (*P* = 0.002) was observed with the use of MiECC.

**Figure 3:**
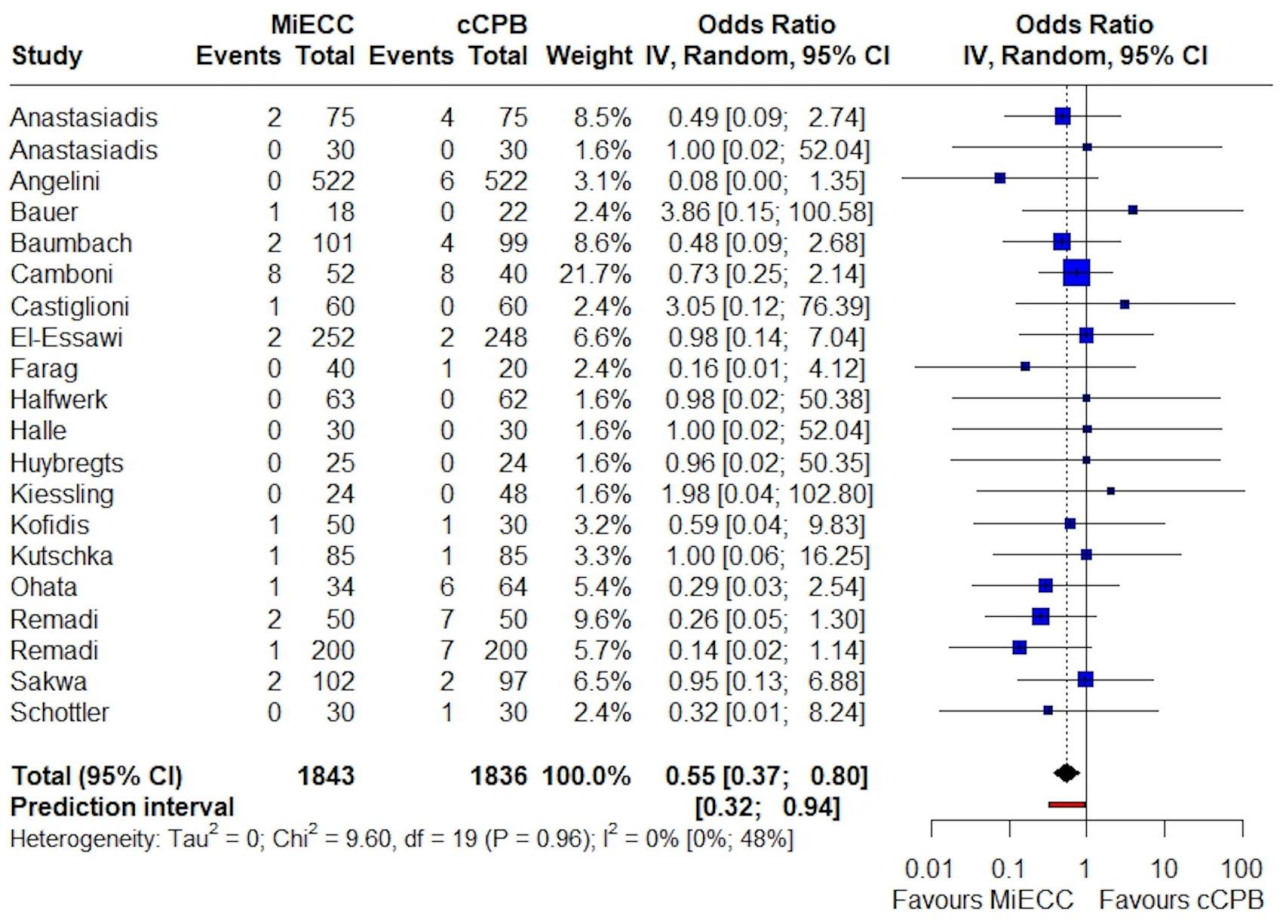
Forest plot of randomized trials comparing the incidence of postoperative cerebrovascular events in patients operated with minimally invasive extracorporeal circulation (MiECC) versus conventional cardiopulmonary bypass (cCPB). A significant reduction was observed with the use of MiECC (*P* = 0.007).

Regarding major morbidity, the incidence of postoperative myocardial infarction was significantly lower in patients operated on MiECC (11 studies; OR 0.42; 95% CI 0.26–0.68; *P* = 0.002; *I*^2^ = 0%) (Fig. 2). There was no evidence of publication bias or small-study effect (Supplementary Material, Fig. S8). As far as the analysis of laboratory values indicative of myocardial damage is concerned, these showed that CK-MB release was significantly increased in cCPB [8 studies; WMD = −9.21 (−16.1, −2.3); *P* = 0.01; *I*^2^ = 84%)] (Supplementary Material, Fig. S9), while no difference was observed in peak cardiac troponin release [9 studies; WMD = −2.27 (−5.25, 0,72); *P* = 0.11, *I*^2^ = 100%)] (Supplementary Material, Fig. S10). Furthermore, MiECC significantly reduced the incidence of cerebrovascular events (20 studies; OR 0.55; 95% CI 0.37–0.80; *P* = 0.007; *I*^2^ = 0%) (Fig. 3; Supplementary Material, Fig. S11). The incidence of postoperative acute kidney injury was similar between groups (12 studies; OR 0.88; 95% CI 0.56–1.37; *P* = 0.5; *I*^2^ = 0.0%) (Supplementary Material, Fig. S12). The same applied to the need for inotropic support (13 studies; OR 0.89; 95% CI 0.64–1.23; *P* = 0.4; *I*^2^ = 35%), incidence of low cardiac output syndrome (6 studies; OR 0.59; 95% CI 0.13–2.63; *P* = 0.4; *I*^2^ = 20%) and need for intra-aortic balloon pump implantation (7 studies; OR 1.09; 95% CI 0.59–1.99; *P* = 0.7; *I*^2^ = 0%) (Supplementary Material, Figs S13–S15). Postoperative atrial fibrillation was assessed in 14 studies and it was found to be significantly reduced in patients operated on MiECC (OR 0.82; 95% CI 0.69–0.98; *P* = 0.03; *I*^2^ = 0%) (Supplementary Material, Fig. S16). The beneficial effect of MiECC was also reflected in the duration of mechanical ventilation [17 studies; WMD = −2.19 (−3.53, −0.86); *P* = 0.003; *I*^2^ = 88%)] (Supplementary Material, Fig. S17) leading to significantly reduced ICU stay [20 studies; WMD = −7.36 (−14.2, −0.54); *P* = 0.01; *I*^2^ = 98%)] (Supplementary Material, Fig. S18) as well as hospital stay [17 studies; WMD = −0.66 (−1.31, −0.01); *P* = 0.02; *I*^2^ = 93%)] (Supplementary Material, Fig. S19).

**Figure 4:**
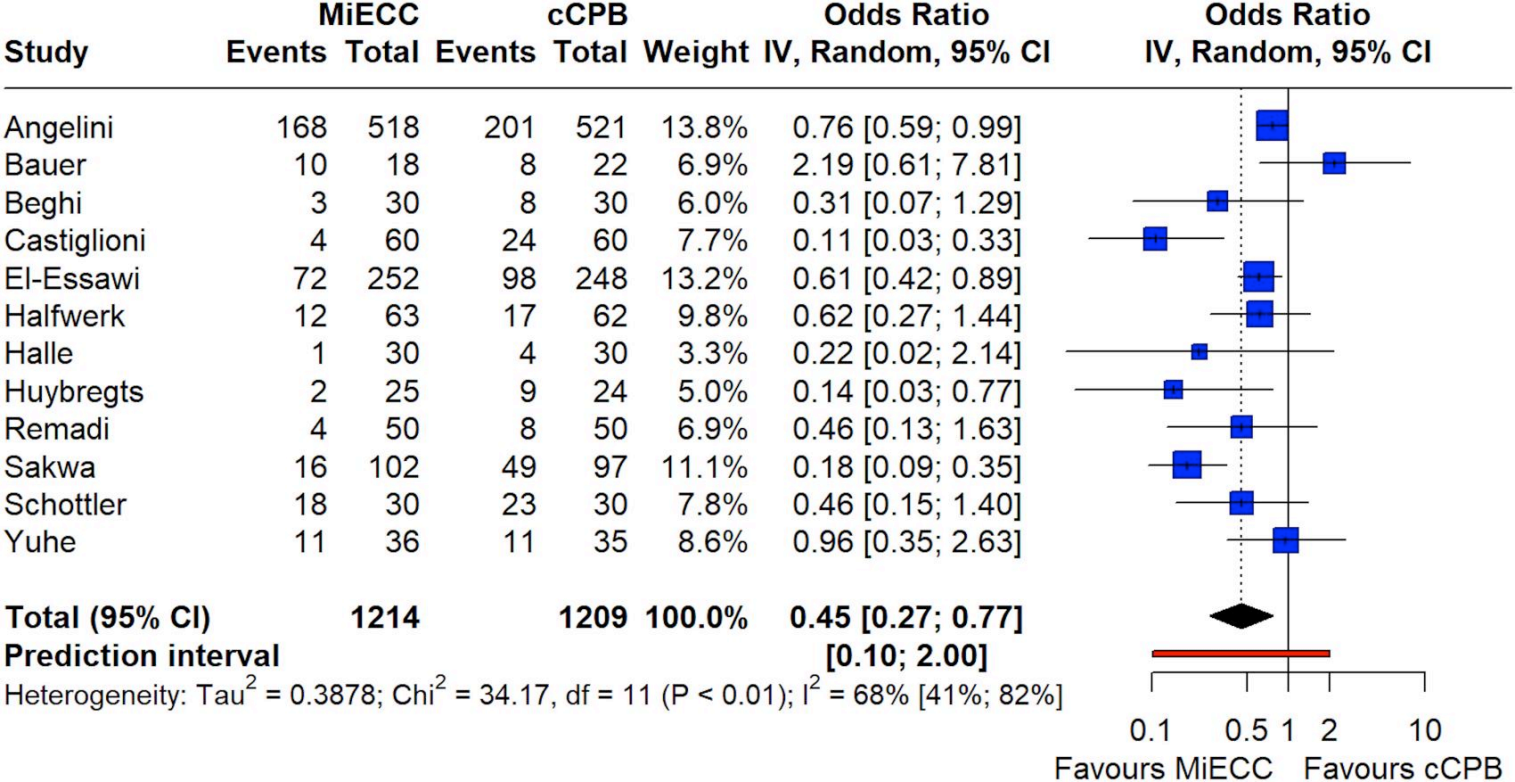
Forest plot of randomized trials comparing the incidence of red blood cells (RBC) transfusion in patients operated with minimally invasive extracorporeal circulation (MiECC) versus conventional cardiopulmonary bypass (cCPB). A significant reduction in the rate of blood transfusion (*P* = 0.006) was observed with the use of MiECC.

Regarding blood conservation, MiECC significantly reduced need for RBC transfusion (12 studies; OR 0.45; 95% CI 0.27–0.77; *P* = 0.006; *I*^2^ = 68%) (Fig. 4). There was no evidence of publication bias or small-study effect (Supplementary Material, Fig. S20). This finding was associated with significantly reduced blood loss [WMD = −144.2 (−208, −80); *P* < 0.001; *I*^2^ = 96%)] (Supplementary Material, Fig. S21) and rate of re-exploration for bleeding (15 studies; OR 0.63; 95% CI 0.42–0.92; *P* = 0.1; *I*^2^ = 0%) in patients operated on MiECC (Supplementary Material, Fig. S22). Moreover, FFP transfusion was significantly reduced when using MiECC [9 studies; WMD = −0.5 (−1.01, 0); *P* < 0.001; *I*^2^ = 91%)] (Supplementary Material, Fig. S23), while preservation of platelet count postoperatively was also favouring MiECC [8 studies; WMD = 33.2 (10.01, 56,5); *P* = 0.01; *I*^2^ = 94%)] (Supplementary Material, Fig. S24). Haemodilution, as calculated by haematocrit drop after CPB, was found to be reduced in MiECC group [6 studies; WMD = 1.95 (−0.9, 4.81); *P* = 0.058; *I*^2^ = 94%)] (Supplementary Material, Fig. S25). Regarding systemic inflammatory markers, MiECC was associated with significantly reduced levels of postoperative PMNE levels [4 studies; WMD = −131.5 (−185.3, 77.8); *P* = 0.008; *I*^2^ = 14%)] (Supplementary Material, Fig. S26), while this trend was not evident in the other biochemical markers CRP [8 studies; WMD = −0.93 (−23.8, 21.9); *P* = 0.9, *I*^2^ = 96%)] (Supplementary Material, Fig. S27) and IL-6 [5 studies; WMD = −39.9 (−107, 27.2), *P* = 0.17, *I*^2^ = 99%)] (Supplementary Material, Fig. S28).

## DISCUSSION

This is the largest published meta-analysis of 36 randomized trials with a total of 4849 patients comparing MiECC to cCPB. The findings of our study demonstrate that MiECC significantly reduces postoperative mortality as well as the incidence of serious adverse events, including postoperative myocardial infarction and stroke. Moreover, MiECC is associated with reduced incidence of postoperative atrial fibrillation, RBC transfusion requirements, blood loss and reoperation rates. This translates into significantly reduced need for mechanical ventilation and duration of ICU and hospital stay. These findings are consistent over time and lie in accordance with results of our previous and other published meta-analyses [5–7].

There is an abundance of existing studies in the literature that consistently indicate superiority of MiECC over cCPB and any other optimized CPB system in major clinical end-points [5–7, 50]. This is attributed mainly to the integral characteristics of MiECC, as defined in the 2016 MiECTiS consensus paper [4]. The diversity of the systems used was a main criticism against previously published trials and meta-analyses [8]. The major methodologic characteristic that differentiates our study from all other published meta-analyses up-to-date is the inclusion of MiECC cohort as defined by MiECTiS consensus paper. This strategy precluded RCTs using MiECC with non-coated biocompatible surfaces, which have been consistently included in previous meta-analyses [12–14]. Moreover, special emphasis was given to the detection of duplicate studies [49, 51]. Despite the significant time frame of included studies spanning 22 years, only 1 study by Baumbach *et al.* involved minimally invasive valve procedures [37]. The vast majority was CABG and conventional valve procedure. Thus, no evolution in surgical and anaesthetic technique as well as in ICU management was anticipated, which could potentially influence results.

The findings of our study lie in agreement with the results from the recently published COMICS trial which showed a 27% relative reduction (*P* = 0.025) in the risk of serious adverse events, as captured by the composite primary outcome, alongside with a significant improvement in patient-reported quality of life with MiECC [8]. Our study is the largest meta-analysis with robust methodology rendering it credible and, hence, providing compelling evidence (class I, level of evidence A) to support the adoption of MiECC as a primary perfusion strategy in contemporary cardiac surgery. The finding that mortality benefit is attributed predominantly to CABG procedures could be explained by the significantly higher proportion of patients operated for CABG (78.3%) compared to other procedures (21.7%). The consistent finding that an advanced perfusion technique, such as MiECC, represents a major contributing factor to an optimal postoperative outcome highlights the concept that ‘perfusion matters, and it will always matter in cardiac surgery’, irrespective of ongoing advancements in surgical techniques [52].

The significant protective hematologic effects of MiECC, as evidenced in our study, have been consistently confirmed by multiple studies [53–56]. Implementation of MiECC has been integrated as an intraoperative strategy for maintenance of haemostasis and blood conservation management in contemporary patient blood management (PBM) protocols [57, 58]. Despite consistent evidence, recently published EACTS/EACTAIC guidelines for PBM still provide a class IIa level of evidence B recommendation for MiECC as a strategy to reduce transfusion and bleeding basing their conclusion on heterogeneity among studies and diversity of outcomes [59]. Results of the present meta-analysis provide robust evidence that MiECC should be upgraded to a class I level of evidence A PBM strategy.

Furthermore, reduced time on mechanical ventilation attained with MiECC, leading to reduced ICU stay and ultimately hospital stay, could facilitate application of modern enhanced recovery in cardiac surgery protocols [60]. Since global healthcare policy authorities combine treatment effectiveness with quality of life and cost effectiveness, the results of this study indicate that the ‘smoother’ postoperative course achieved with MiECC could exert a major financial benefit on the healthcare system by reducing the resources used, while increasing the satisfaction of the patients [61, 62].

### Limitations

There are several limitations to this study. The main limitation of this meta-analysis lies within the methodologic variability of the MiECC and cCPB systems used in each study, as evidenced in Table 2. Differences in MiECC type, circuit components, anticoagulation strategy, cardioplegia, circuit coating as well as priming volumes could impact the clinical outcomes measured. Moreover, as the time frame of included studies reached 22 years, there were no homogenous definitions for the outcomes measured between studies, especially postoperative myocardial infarction, stroke and acute kidney injury. The quality of the studies also varied. Several studies showed a considerable risk of bias. Regarding the primary outcome, the 1st domain of RoB2 tool raised the majority of concerns mainly due to lacking reporting of the randomization process and the allocation to treatment. Moreover, concerns were raised for most of the included studies in the 5th domain, mainly due to the lack of a study protocol or an analysis plan. Moreover, the wide prediction intervals for certain outcomes, such as ICU and hospital stay, reflect significant between-study heterogeneity. This suggests that while the pooled effect size demonstrates statistical significance, the true effect in individual future studies may vary widely. Such variability underscores the need for cautious interpretation of these findings particularly in settings with differing MiECC systems and procedural practices.

## CONCLUSION

This meta-analysis provides an updated comparison of MiECC versus cCPB in a series of adverse postoperative clinical outcomes. Supporting previously reported evidence, MiECC demonstrated a significant overall survival benefit, attributed mainly to CABG procedures. Moreover, MiECC reduced significantly the incidence of postoperative complications including myocardial infarction, cerebrovascular events and atrial fibrillation while promoting an effective blood conservation strategy by significantly reducing transfusion requirements, blood loss and need for re-exploration. The overall benefit was reflected in reduced ICU and hospital stay. In the era when the cardiac surgical community urges for minimal invasiveness and optimum results in terms of enhancing patient recovery, especially in high-risk and complex cases, this meta-analysis provides robust evidence for the beneficial effect of MiECC for the patient as well as for the healthcare system.

## Supplementary Material

ezaf112_Supplementary_Data

## Data Availability

The data underlying this article will be shared on reasonable request to the corresponding author.

## ABBREVIATIONS

AVR: Aortic valve replacement
CABG: Coronary artery bypass grafting
CPB: Cardiopulmonary bypass
cCPB: Conventional cardiopulmonary bypass
EACTAIC: European Association of Cardiothoracic Anaesthesiology and Intensive Care
EACTS: European Association for Cardio-Thoracic Surgery
FFP: Fresh frozen plasma
ICU: Intensive care unit
MiECC: Minimal invasive extracorporeal circulation
MiECTiS: Minimal Invasive Extracorporeal Technologies International Society
OR: Odds ratio
PBM: Patient blood management
PRISMA: Preferred Reporting Items for Systematic Reviews and Meta-Analyses
PROSPERO: Prospective Register of Systematic Reviews in Health and Social Care
RBC: Red blood cell
RCTs: Randomized controlled trials
RoB2: Risk of bias 2 tool
WMD: Weighted mean difference
