# Supplementary material for: Minimal invasive extracorporeal circulation versus conventional cardiopulmonary bypass in cardiac surgery: a contemporary systematic review and meta-analysis
Source: Eur J Cardiothorac Surg. 2025 Mar 25;67(4):ezaf112. doi: 10.1093/ejcts/ezaf112 (PMC11985097; doi:10.1093/ejcts/ezaf112)
Supplement: ezaf112_Supplementary_Data [file ezaf112_supplementary_data.pdf]

Supplementary material

Supplementary Table 1. Database search strategy

**Search terms**
“minimized extracorporeal circulation” or “mini extracorporeal circulation” or “minimal extracorporeal circulation” or “minimal invasive extracorporeal circulation” or “miniaturized extracorporeal circulation” or “minimizing extracorporeal circulation” or “minimally invasive extracorporeal circulation” “minimal invasive extracorporeal circuits” or “minimal invasive extracorporeal systems” or “mini cardiopulmonary bypass” or “minimal cardiopulmonary bypass” or “minimized cardiopulmonary bypass” or “miniaturized cardiopulmonary bypass” or “minimizing cardiopulmonary bypass” or “closed circuit extracorporeal circulation” or “closed circuit cardiopulmonary bypass” or “Medtronic resting heart system” or “ECCO(2)R” or “CORx” or “Capiox” or “mini heart lung machine” or “ROCSafe” or “Jostra” or “Jostra-Maquet” or MECC or MiECC or MIECTIS<br><br>Each of the following terms was combined with “coronary artery bypass grafting” or “heart valve surgery”

Supplementary Table 2. Definitions of major outcomes.

Author	Mortality	Postoperative MI	Cerebrovascular events	ARF	Myocardial protection
**Fromes et al.**[15]	N/D	N/D	N/R	N/D	N/R
**Remadi et al.**[16]	30-day	N/D	Aphasia, focal motor, sensory cranial abnormality	N/D	Peak Tn, Inotropic support, LCOS, IABP
**Beghi et al.**[17]	In-hospital	N/D	N/R	Cr level > 2 mg/dl	Peak Tn, inotropic support, LCOS, IABP
**Bical et al.**[18]	In-hospital	N/D	N/R	N/D	N/R
**Remadi et al.**[19]	30-day	N/D	Aphasia, focal motor, sensory cranial abnormality	N/D	Peak Tn, Inotropic support, LCOS, IABP
**Huybregts et al.**[20]	N/D	N/D	N/D	N/D	N/R
**Skrabal et al.**[21]	N/D	N/D	N/R	N/D	Peak Tn, inotropic support
**Valtonen et al.**[22]	In-hospital	N/D	N/R	N/D	Peak Tn
**Perthel et al.**[23]	N/D	N/D	N/R	N/D	N/R
**Kofidis et al.**[24]	In-hospital	N/D	Stroke	N/D	Peak Tn
**Ohata et al.**[25]	N/D	N/D	Brain death, nonfatal stroke, transient ischemic attack	Cr level > 2 mg/dl	N/R
**Schöttler et al.**[26]	In-hospital	N/D	Stroke	N/D	Peak Tn, inotropic support
**Castiglioni et al.**[27]	In-hospital	N/D	Major neurological events	N/D	Peak Tn, inotropic support, IABP
**Kutschka et al.**[28]	N/D	N/D	Stroke	N/D	Inotropic support
**Gunaydin et al.**[29]	N/D	N/D	N/R	N/D	Inotropic support, IABP
**Sakwa et al.**[30]	N/D	N/D	Cerebrovascular accidents	N/D	IABP
**Camboni et al.**[31]	30-day	N/D	Neurological dysfunction	N/D	N/R
**Anastasiadis et al.**[32]	In-hospital	N/D	N/D	N/D	N/R
**Bauer et al.**[33]	30-day	N/D	Stroke, TIA	N/D	Inotropic support
**El-Essawi et al.**[34]	In-hospital	N/D	Stroke	N/R	Peak Tn, inotropic support
**Abdel Aal et al.**[35]	N/D	N/D	N/R	N/R	Inotropic support
**Anastasiadis et al.**[36]	3-month	CK-MB levels > 125 IU/L during the first 72 hours plus new pathologic Q waves or LBBB	N/D	Cr level > 2 mg/dl or doubling of pre-op levels	N/R
**Baumbach et al.**[37]	In-hospital	N/D	Apoplexy, TIA	Dialysis	N/R
**Deininger et al.**[38]	N/D	N/D	N/R	N/D	N/R

**Anastasiadis et al.**[39]	In-hospital	CK-MB levels>125 IU/L during the first 72 hours plus new pathologic Q waves or LBBB	Neurologic deficit of abrupt onset persisting > 24h	Cr level > 2 mg/dl or doubling of pre-op levels	Peak Tn
**Farag et al.**[40]	In-hospital	N/D	Stroke	N/D	Peak Tn
**Kiessling et al.**[41]	30-day	STEMI or NSTEMI <7 days ago	N/D	N/D	Inotropic support, LCOS
**Elci et al.**[42]	N/D	N/D	N/R	N/D	Peak Tn, inotropic support
**Halfwerk et al.**[43]	30-day	CK-MB values 10 times CK and new alterations in ECG or TTE	Stroke	eGFR reduction > 50% within 48h	Peak Tn
**Yuhe et al.**[44]	N/D	N/D	Stroke	N/R	N/R
**Media et al.**[45]	N/D	N/D	N/R	AKIN 1-3	N/R
**Condello et al.**[46]	N/D	N/D	N/R	N/R	N/R
**Gunaydin et al.**[47]	N/D	N/D	N/R	N/R	N/R
**Ellam et al.**[48]	N/D	N/D	N/R	N/R	Inotropic support
**Angelini et al.**[8]	30-day	serum Tn concentrations and ECG recording	Stroke (brain imaging by CT or MRI, in association with new onset focal or generalized neurological deficit)	AKIN 3, dialysis	Inotropic support, LCOS, IABP
**Halle et al.**[49]	N/D	N/D	N/R	N/R	Inotropic support, LCOS

AKIN: Acute kidney injury network; ARF: Acute renal failure; Cr: Creatinine; ECG: Electrocardiogram; LBBB: Left bundle branch block; IABP: Intra-aortic balloon pump; LCOS: Low cardiac output syndrome; MI: Myocardial infarction; N/D: Not defined; N/R: Not recorded; NSTEMI: Non ST-elevation myocardial infarction; STEMI: ST-elevation myocardial infarction; TIA: Transient ischemic attack; Tn: Troponin.

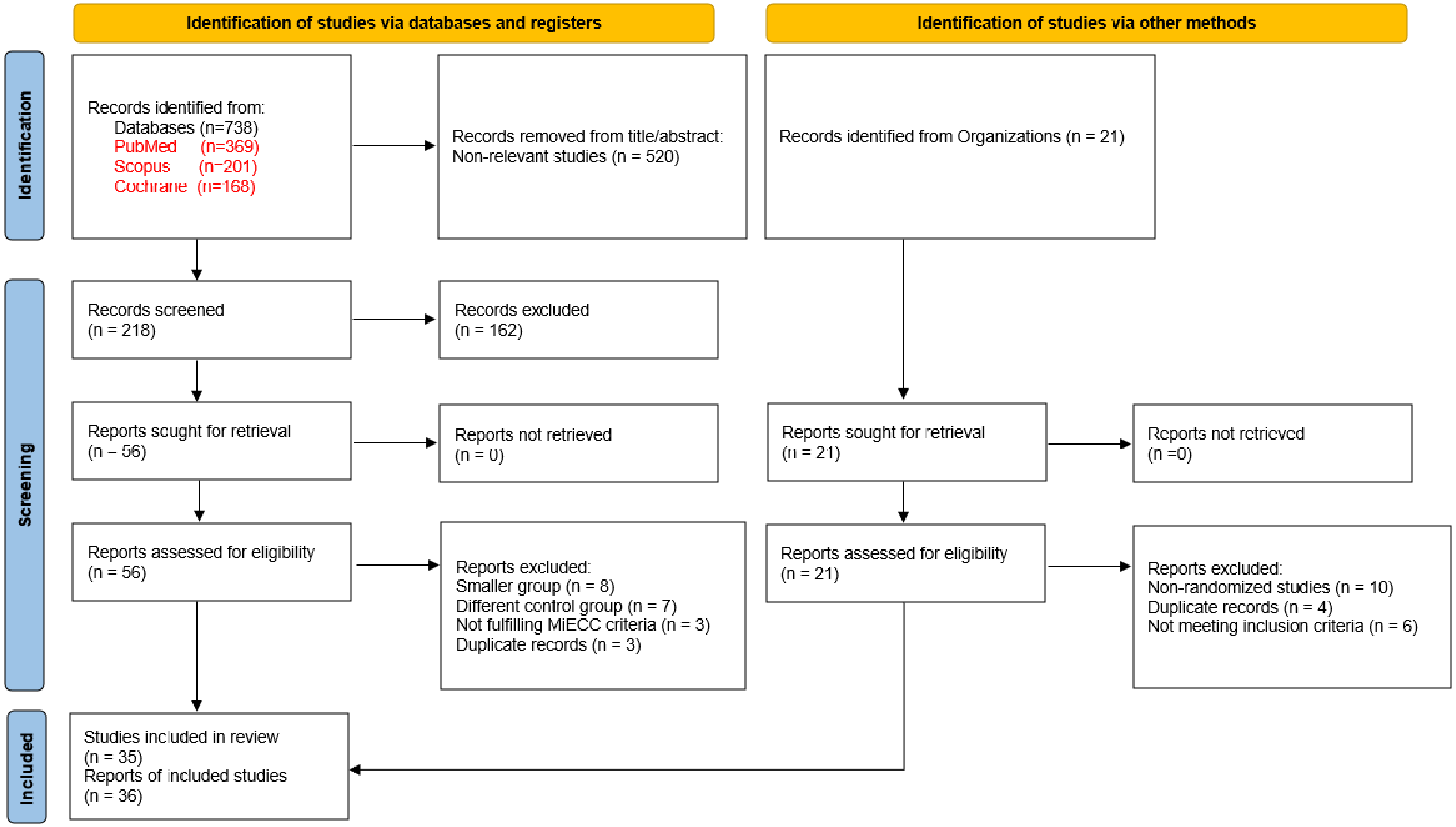

Supplementary Figure 1. PRISMA flow diagram

Study	MiECC Mean	MiECC SD	MiECC Total	cCPB Mean	cCPB SD	cCPB Total	Weight	Mean Difference IV, Random, 95% CI
Anastasiadis	103.00	25.0000	50	128.00	44.0000	49	2.3%	-25.00 [-39.13; -10.87]
Anastasiadis	102.90	25.0000	75	122.20	33.0000	75	3.3%	-19.30 [-28.67; -9.93]
Anastasiadis	86.80	45.1000	30	96.60	25.2000	30	1.6%	-9.80 [-28.29; 8.69]
Angelini	89.30	30.1000	535	87.30	30.4000	536	4.8%	2.00 [-1.62; 5.62]
Bauer	72.00	17.0000	18	76.00	20.0000	22	2.8%	-4.00 [-15.47; 7.47]
Baumbach	102.24	44.3100	101	94.28	42.7700	99	2.7%	7.96 [-4.11; 20.03]
Beghi	99.00	28.0000	30	78.00	19.0000	30	2.7%	21.00 [8.89; 33.11]
Bical	79.00	14.0000	20	79.00	13.0000	20	3.6%	0.00 [-8.37; 8.37]
Camboni	88.70	24.0000	52	97.50	30.6000	40	2.8%	-8.80 [-20.31; 2.71]
Castiglioni	69.00	10.0000	60	65.00	8.5000	60	4.9%	4.00 [0.68; 7.32]
Condello	62.00	15.2000	30	60.00	7.3600	30	4.2%	2.00 [-4.04; 8.04]
Deininger	112.00	.	36	115.00	.	39	0.0%	-3.00
Elci	85.85	20.5600	31	84.92	19.7500	27	3.1%	0.93 [-9.46; 11.32]
El-Essawi	74.90	26.7000	252	78.50	35.8000	248	4.3%	-3.60 [-9.14; 1.94]
Ellam	93.70	30.4000	120	89.50	28.5000	120	3.8%	4.20 [-3.26; 11.66]
Fromes	61.00	20.0000	30	79.00	22.0000	30	3.0%	-18.00 [-28.64; -7.36]
Gunaydin	98.00	4.0000	20	94.50	4.0000	20	5.0%	3.50 [1.02; 5.98]
Halfwerk	74.00	18.0000	63	80.00	26.0000	62	3.7%	-6.00 [-13.85; 1.85]
Halle	87.00	25.0000	30	76.00	27.0000	30	2.4%	11.00 [-2.17; 24.17]
Huybregts	95.00	4.0000	25	100.00	3.0000	24	5.1%	-5.00 [-6.97; -3.03]
Kiessling	115.00	49.0000	24	103.00	30.0000	48	1.3%	12.00 [-9.36; 33.36]
Kofidis	74.00	17.0000	50	82.00	24.0000	30	3.2%	-8.00 [-17.80; 1.80]
Kutschka	103.00	37.9000	85	106.90	44.9000	85	2.6%	-3.90 [-16.39; 8.59]
Media	87.00	.	30	76.00	.	30	0.0%	11.00
Ohata	146.00	35.0000	34	147.00	44.0000	64	2.0%	-1.00 [-16.96; 14.96]
Remadi	71.00	23.0000	50	77.00	27.0000	50	3.2%	-6.00 [-15.83; 3.83]
Remadi	63.00	19.0000	200	65.00	18.0000	200	4.8%	-2.00 [-5.63; 1.63]
Sakwa	75.00	20.0000	102	76.00	20.0000	97	4.3%	-1.00 [-6.56; 4.56]
Schottler	103.00	27.0000	30	101.00	28.0000	30	2.3%	2.00 [-11.92; 15.92]
Skrabal	85.00	3.0000	30	92.00	4.0000	30	5.1%	-7.00 [-8.79; -5.21]
Valtonen	77.50	19.0000	20	85.00	21.0000	20	2.6%	-7.50 [-19.91; 4.91]
Yuhe	102.90	33.1000	36	97.30	17.6000	35	2.6%	5.60 [-6.68; 17.88]
**Total (95% CI)**			**2429**			**2420**	**100.0%**	**-2.02 [-5.17; 1.13]**
**Prediction interval**								**[-15.29; 11.24]**

Heterogeneity: $Tau^2 = 39.8547$; $Chi^2 = 140.54$, df = 29 (P < 0.01); $I^2 = 79\%$ [71%; 85%]

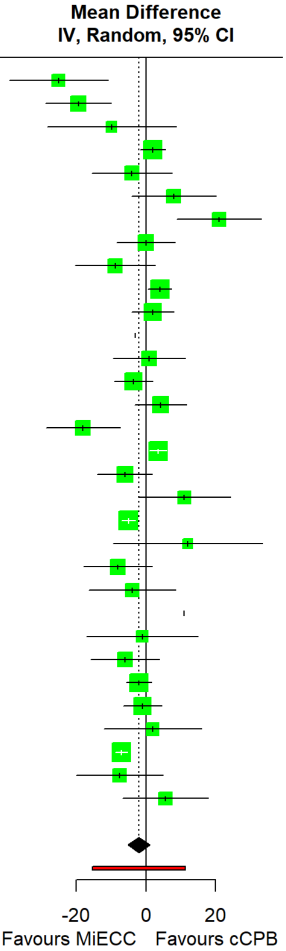

Supplementary Figure 2. Forest plot of randomized trials comparing cardiopulmonary bypass time in patients operated with minimal invasive extracorporeal circulation (MiECC) versus conventional cardiopulmonary bypass (cCPB). A significant reduction (p<0.001) is observed with MiECC.

Study	MiECC Mean	MiECC SD	MiECC Total	cCPB Mean	cCPB SD	cCPB Total	Weight	Mean Difference IV, Random, 95% CI
Anastasiadis	65.00	17.0000	50	72.00	22.0000	49	2.6%	-7.00 [-14.76; 0.76]
Anastasiadis	64.80	17.6000	75	68.60	17.2000	75	3.7%	-3.80 [-9.37; 1.77]
Anastasiadis	55.20	29.6000	30	61.20	14.3000	30	1.4%	-6.00 [-17.76; 5.76]
Angelini	57.00	21.0000	535	56.40	21.4000	536	5.8%	0.60 [-1.94; 3.14]
Bauer	40.00	11.0000	18	41.00	15.0000	22	2.5%	-1.00 [-9.07; 7.07]
Baumbach	66.60	30.5400	101	61.09	21.6200	99	2.8%	5.51 [-1.81; 12.83]
Beghi	59.00	20.0000	30	45.00	13.0000	30	2.3%	14.00 [5.46; 22.54]
Bical	61.00	13.0000	20	62.00	12.0000	20	2.6%	-1.00 [-8.75; 6.75]
Camboni	53.00	17.4000	52	63.20	21.3000	40	2.4%	-10.20 [-18.32; -2.08]
Castiglioni	54.00	12.5000	60	49.00	17.0000	60	3.8%	5.00 [-0.34; 10.34]
Condello	42.00	9.0000	30	43.00	7.0000	30	4.7%	-1.00 [-5.08; 3.08]
Deininger	55.00	.	36	58.00	.	39	0.0%	-3.00
Elci	36.41	9.4100	31	35.74	11.4900	27	3.8%	0.67 [-4.78; 6.12]
El-Essawi	48.20	20.5000	252	49.90	22.5000	248	4.9%	-1.70 [-5.47; 2.07]
Ellam	80.30	25.8000	120	77.70	23.8000	120	3.3%	2.60 [-3.68; 8.88]
Farag	51.94	7.2400	40	47.17	3.2000	20	5.7%	4.77 [2.12; 7.42]
Fromes	63.00	17.0000	30	56.00	15.0000	30	2.5%	7.00 [-1.11; 15.11]
Gunaydin	77.00	3.0000	20	74.50	3.0000	20	6.2%	2.50 [0.64; 4.36]
Halfwerk	51.00	17.0000	63	54.00	20.0000	62	3.2%	-3.00 [-9.51; 3.51]
Halle	47.00	22.0000	30	45.00	26.0000	30	1.4%	2.00 [-10.19; 14.19]
Huybregts	71.00	3.0000	25	68.00	3.0000	24	6.3%	3.00 [1.32; 4.68]
Kofidis	42.00	12.0000	50	46.00	11.0000	30	4.0%	-4.00 [-9.15; 1.15]
Kutschka	76.50	29.5000	85	79.00	34.0000	85	2.0%	-2.50 [-12.07; 7.07]
Media	47.00	.	30	45.00	.	30	0.0%	2.00
Ohata	93.00	28.0000	34	107.00	34.0000	64	1.3%	-14.00 [-26.57; -1.43]
Remadi	40.00	20.0000	50	46.00	17.0000	50	2.8%	-6.00 [-13.28; 1.28]
Remadi	31.00	12.0000	200	33.00	9.5000	200	6.1%	-2.00 [-4.12; 0.12]
Schottler	61.00	19.0000	30	61.00	18.0000	30	2.0%	0.00 [-9.37; 9.37]
Skrabal	52.00	2.5000	30	49.50	3.0000	30	6.5%	2.50 [1.10; 3.90]
Valtonen	58.00	17.0000	20	65.00	19.0000	20	1.6%	-7.00 [-18.17; 4.17]
Yuhe	58.20	26.3000	36	51.40	11.3000	35	2.0%	6.80 [-2.57; 16.17]
**Total (95% CI)**			**2429**			**2420**	**100.0%**	**0.21 [-1.61; 2.02]**
**Prediction interval**								**[-6.39; 6.80]**

Heterogeneity: $Tau^2 = 9.6800$; $Chi^2 = 78.79$, df = 28 ($P < 0.01$); $I^2 = 64\%$ [47%; 76%]

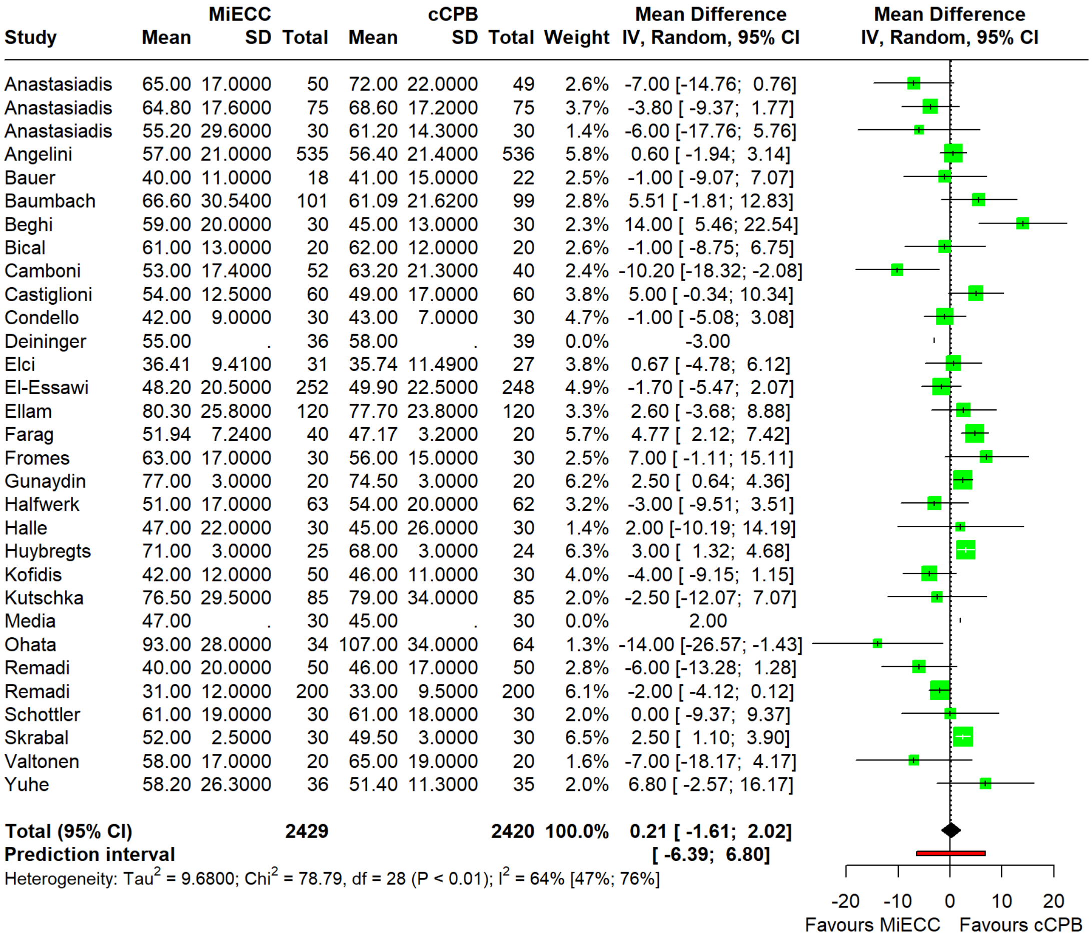

Supplementary Figure 3. Forest plot of randomized trials comparing aortic cross-clamp time in patients operated with minimal invasive extracorporeal circulation (MiECC) versus conventional cardiopulmonary bypass (cCPB). No difference is observed between groups.

Study or Subgroup	MiECC Events	MiECC Total	cCPB Events	cCPB Total	Weight	Odds Ratio IV, Random, 95% CI
proc_bin = Various						
Remadi	1	50	2	50	3.6%	0.49 [0.04; 5.58]
Bical	0	20	0	20	1.4%	1.00 [0.02; 52.85]
Castiglioni	0	60	0	60	1.4%	1.00 [0.02; 51.22]
Kutschka	0	85	0	85	1.4%	1.00 [0.02; 50.98]
El-Essawi	1	252	3	248	4.2%	0.33 [0.03; 3.15]
Baumbach	1	101	3	99	4.1%	0.32 [0.03; 3.13]
Halfwerk	0	63	0	62	1.4%	0.98 [0.02; 50.38]
Angelini	3	83	0	82	2.4%	7.17 [0.36; 141.11]
Total (95% CI)		714		706	19.9%	0.70 [0.29; 1.67]
Heterogeneity: $Tau^2 = 0$; $Chi^2 = 3.44$, df = 7 (P = 0.84); $I^2 = 0\%$ [0%; 68%]						
proc_bin = CABG						
Fromes	0	30	0	30	1.4%	1.00 [0.02; 52.04]
Beghi	0	30	0	30	1.4%	1.00 [0.02; 52.04]
Remadi	3	200	5	200	10.3%	0.59 [0.14; 2.52]
Huybregts	0	25	0	24	1.4%	0.96 [0.02; 50.35]
Skrabal	0	30	0	30	1.4%	1.00 [0.02; 52.04]
Kofidis	0	50	1	30	2.1%	0.19 [0.01; 4.94]
Ohata	1	34	5	64	4.5%	0.36 [0.04; 3.19]
Schottler	0	30	0	30	1.4%	1.00 [0.02; 52.04]
Gunaydin	1	20	2	20	3.5%	0.47 [0.04; 5.69]
Sakwa	0	102	0	97	1.4%	0.95 [0.02; 48.41]
Camboni	0	52	3	40	2.4%	0.10 [0.01; 2.03]
Anastasiadis	1	50	2	49	3.6%	0.48 [0.04; 5.47]
Bauer	0	18	0	22	1.4%	1.22 [0.02; 64.31]
Anastasiadis	3	75	4	75	9.2%	0.74 [0.16; 3.42]
Anastasiadis	0	30	1	30	2.0%	0.32 [0.01; 8.24]
Deininger	0	36	0	39	1.4%	1.08 [0.02; 55.96]
Farag	0	40	0	20	1.4%	0.51 [0.01; 26.44]
Kiessling	1	24	0	48	2.1%	6.19 [0.24; 157.82]
Elci	0	31	0	27	1.4%	0.87 [0.02; 45.48]
Media	0	30	0	30	1.4%	1.00 [0.02; 52.04]
Yuhe	0	36	0	35	1.4%	0.97 [0.02; 50.36]
Condello	0	30	0	30	1.4%	1.00 [0.02; 52.04]
Ellam	0	120	0	120	1.4%	1.00 [0.02; 50.81]
Gunaydin	0	20	0	20	1.4%	1.00 [0.02; 52.85]
Angelini	5	439	10	442	18.4%	0.50 [0.17; 1.47]
Halle	0	30	0	30	1.4%	1.00 [0.02; 52.04]
Total (95% CI)		1612		1612	80.1%	0.61 [0.48; 0.79]
Heterogeneity: $Tau^2 = 0$; $Chi^2 = 5.4$, df = 25 (P = 1.00); $I^2 = 0\%$ [0%; 43%]						
**Total (95% CI)**		**2326**		**2318**	**100.0%**	**0.63 [0.49; 0.81]**
**Prediction interval**						**[0.39; 1.02]**
Heterogeneity: $Tau^2 = 0$; $Chi^2 = 8.89$, df = 33 (P = 1.00); $I^2 = 0\%$ [0%; 39%]						
Test for subgroup differences: $Chi^2 = 0.10$, df = 1 (P = 0.75)						

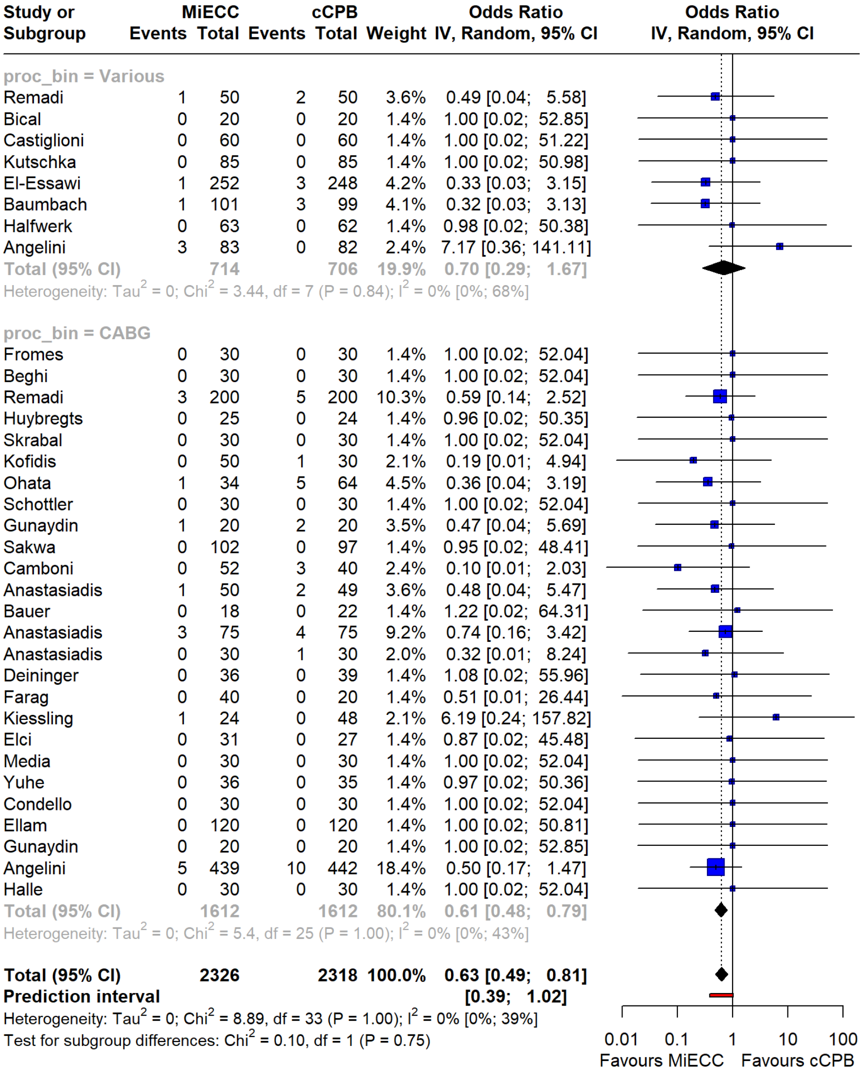

Supplementary Figure 4. Forest plot for mortality comparing subgroups of patients undergoing CABG and other procedures. *cCPB conventional cardiopulmonary bypass; MiECC: Minimal Invasive Extracorporeal Circulation.*

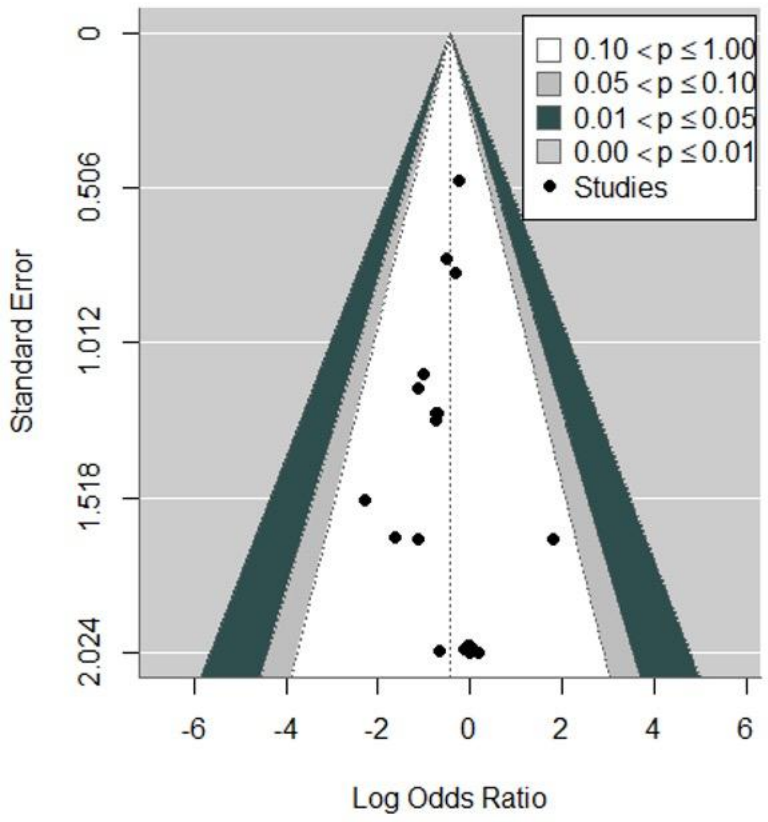

Supplementary Figure 5. Funnel plot for mortality. The funnel plot suggested a distinct imbalance for studies with negative effect and an excess of studies with no effect. This asymmetry could be explained by publication bias (and probably multiple publication bias). No formal tests were deemed appropriate to run due to the number of studies with similar standard errors of the intervention effect estimates.

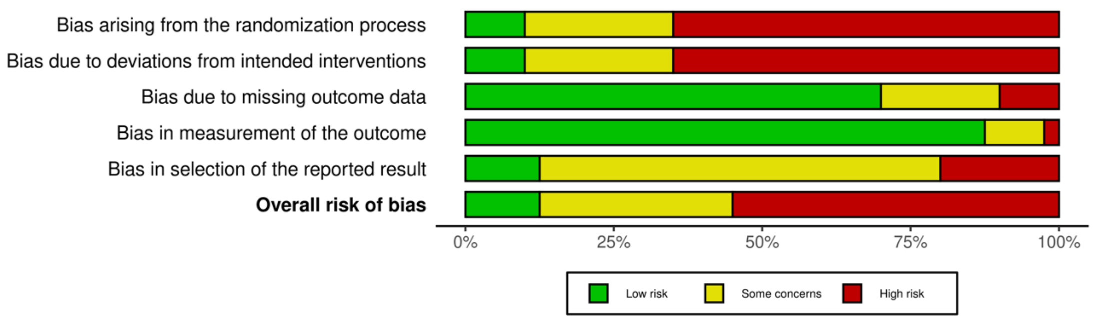

Supplementary Figure 6. Risk of bias graph on the primary outcome (mortality). There is concern regarding the first domain due to lack of reporting of the randomization process and the allocation to treatment. Concerns were raised for most of the included studies in the fifth domain, mainly due to the lack of a study protocol or an analysis plan.

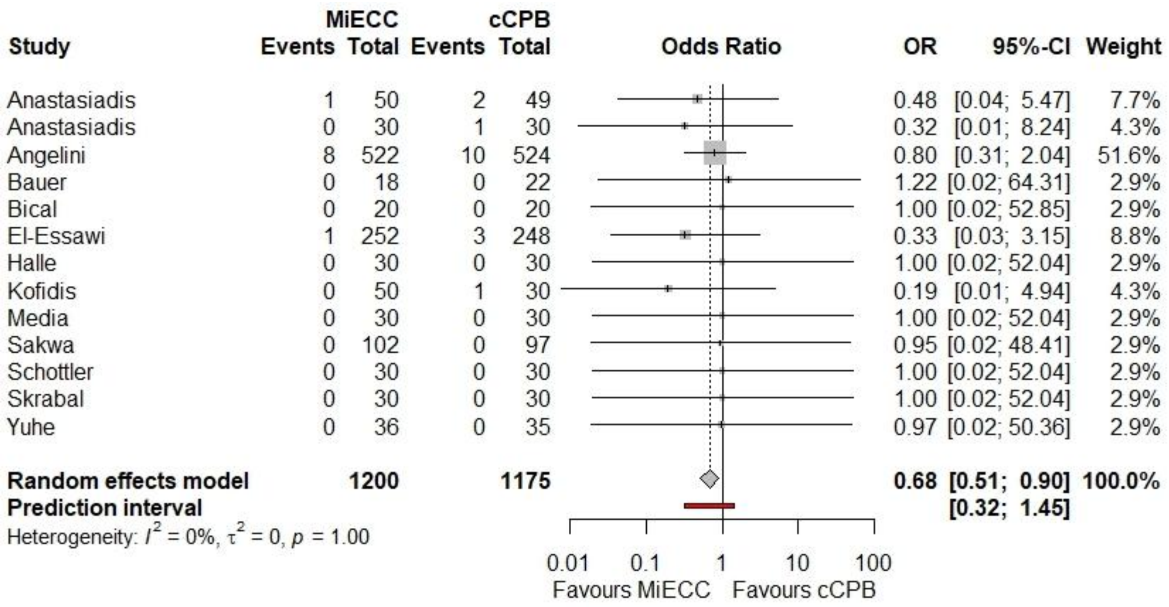

Supplementary Figure 7. Sensitivity analysis excluding studies with overall high risk of bias in RoB2.

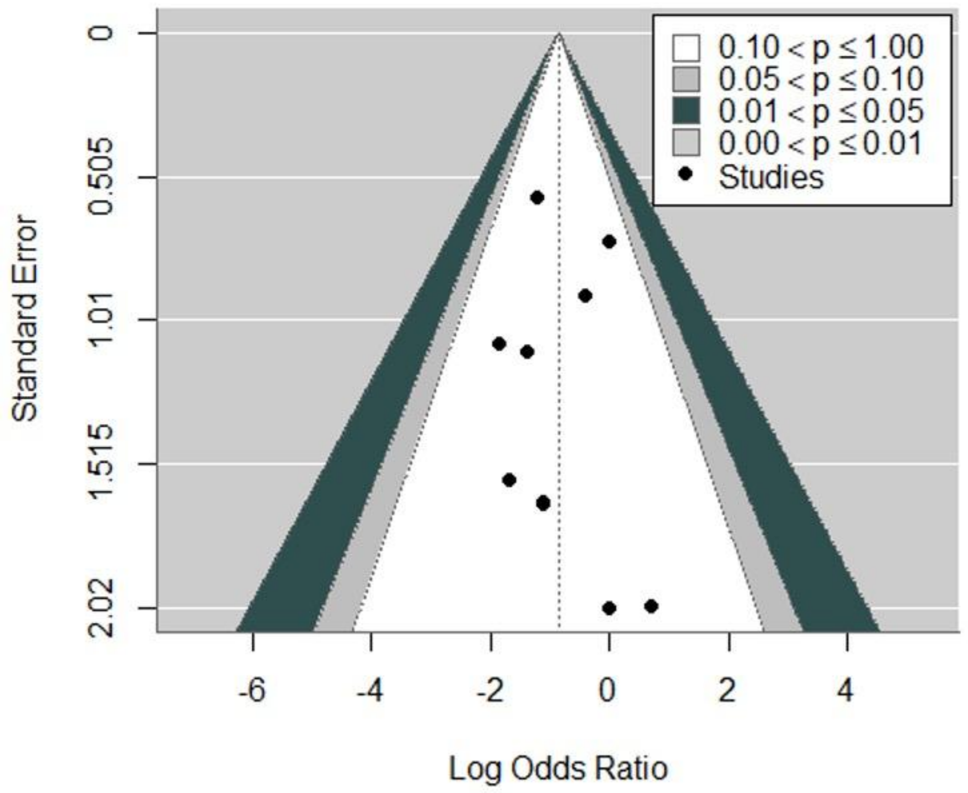

Supplementary Figure 8. Funnel plot for postoperative myocardial infarction. The funnel plot is symmetric. No studies were detected as potential outliers.

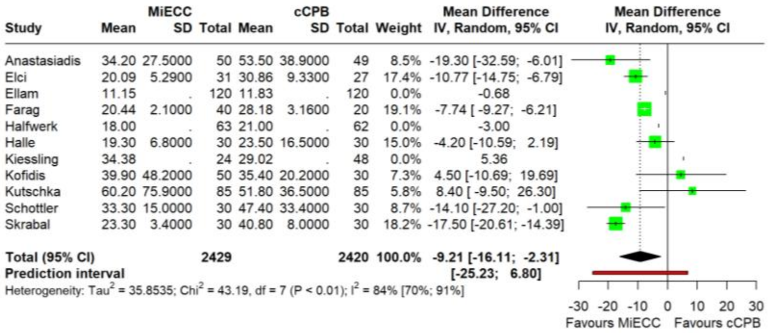

Study	MiECC Mean	MiECC SD	MiECC Total	cCPB Mean	cCPB SD	cCPB Total	Weight	Mean Difference IV, Random, 95% CI
Anastasiadis	34.20	27.5000	50	53.50	38.9000	49	8.5%	-19.30 [-32.59; -6.01]
Elci	20.09	5.2900	31	30.86	9.3300	27	17.4%	-10.77 [-14.75; -6.79]
Ellam	11.15	.	120	11.83	.	120	0.0%	-0.68
Farag	20.44	2.1000	40	28.18	3.1600	20	19.1%	-7.74 [-9.27; -6.21]
Halfwerk	18.00	.	63	21.00	.	62	0.0%	-3.00
Halle	19.30	6.8000	30	23.50	16.5000	30	15.0%	-4.20 [-10.59; 2.19]
Kiessling	34.38	.	24	29.02	.	48	0.0%	5.36
Kofidis	39.90	48.2000	50	35.40	20.2000	30	7.3%	4.50 [-10.69; 19.69]
Kutschka	60.20	75.9000	85	51.80	36.5000	85	5.8%	8.40 [-9.50; 26.30]
Schottler	33.30	15.0000	30	47.40	33.4000	30	8.7%	-14.10 [-27.20; -1.00]
Skrabal	23.30	3.4000	30	40.80	8.0000	30	18.2%	-17.50 [-20.61; -14.39]
**Total (95% CI)**			**2429**			**2420**	**100.0%**	**-9.21 [-16.11; -2.31]**
**Prediction interval**								**[-25.23; 6.80]**

Heterogeneity: $Tau^2 = 35.8535$; $Chi^2 = 43.19$, df = 7 ($P < 0.01$); $I^2 = 84\%$ [70%; 91%]

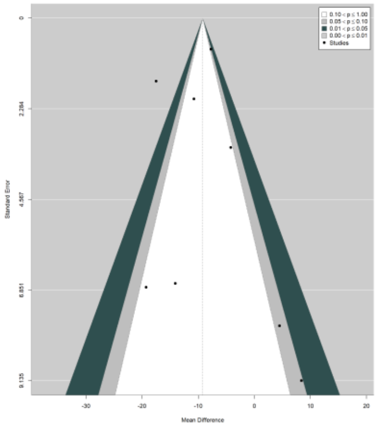

Supplementary Figure 9. (Left image): Forest plot of randomized trials comparing CK-MB release in patients operated with minimal invasive extracorporeal circulation (MiECC) versus conventional cardiopulmonary bypass (cCPB). A significant increase in CK-MB release is observed with cCPB (p=0.01). (Right image): The funnel plot was relatively symmetric (two studies laying outside the contours, symmetrically).

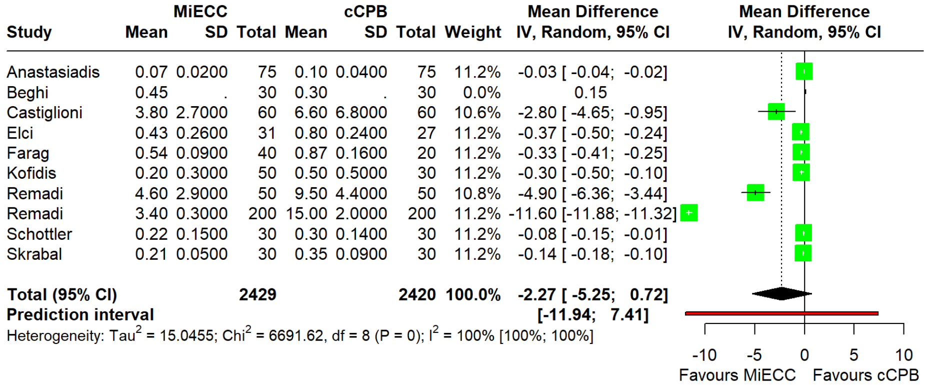

Study	MiECC Mean	MiECC SD	MiECC Total	cCPB Mean	cCPB SD	cCPB Total	Weight	Mean Difference IV, Random, 95% CI
Anastasiadis	0.07	0.0200	75	0.10	0.0400	75	11.2%	-0.03 [-0.04; -0.02]
Beghi	0.45	.	30	0.30	.	30	0.0%	0.15
Castiglioni	3.80	2.7000	60	6.60	6.8000	60	10.6%	-2.80 [-4.65; -0.95]
Elci	0.43	0.2600	31	0.80	0.2400	27	11.2%	-0.37 [-0.50; -0.24]
Farag	0.54	0.0900	40	0.87	0.1600	20	11.2%	-0.33 [-0.41; -0.25]
Kofidis	0.20	0.3000	50	0.50	0.5000	30	11.2%	-0.30 [-0.50; -0.10]
Remadi	4.60	2.9000	50	9.50	4.4000	50	10.8%	-4.90 [-6.36; -3.44]
Remadi	3.40	0.3000	200	15.00	2.0000	200	11.2%	-11.60 [-11.88; -11.32]
Schottler	0.22	0.1500	30	0.30	0.1400	30	11.2%	-0.08 [-0.15; -0.01]
Skrabal	0.21	0.0500	30	0.35	0.0900	30	11.2%	-0.14 [-0.18; -0.10]
**Total (95% CI)**			**2429**			**2420**	**100.0%**	**-2.27 [-5.25; 0.72]**
**Prediction interval**								**[-11.94; 7.41]**

Heterogeneity: $Tau^2 = 15.0455$; $Chi^2 = 6691.62$, df = 8 ($P = 0$); $I^2 = 100\%$ [100%; 100%]

Supplementary Figure 10. Forest plot of randomized trials comparing peak Troponin release in patients operated with minimal invasive extracorporeal circulation (MiECC) versus conventional cardiopulmonary bypass (cCPB). No difference is observed between groups.

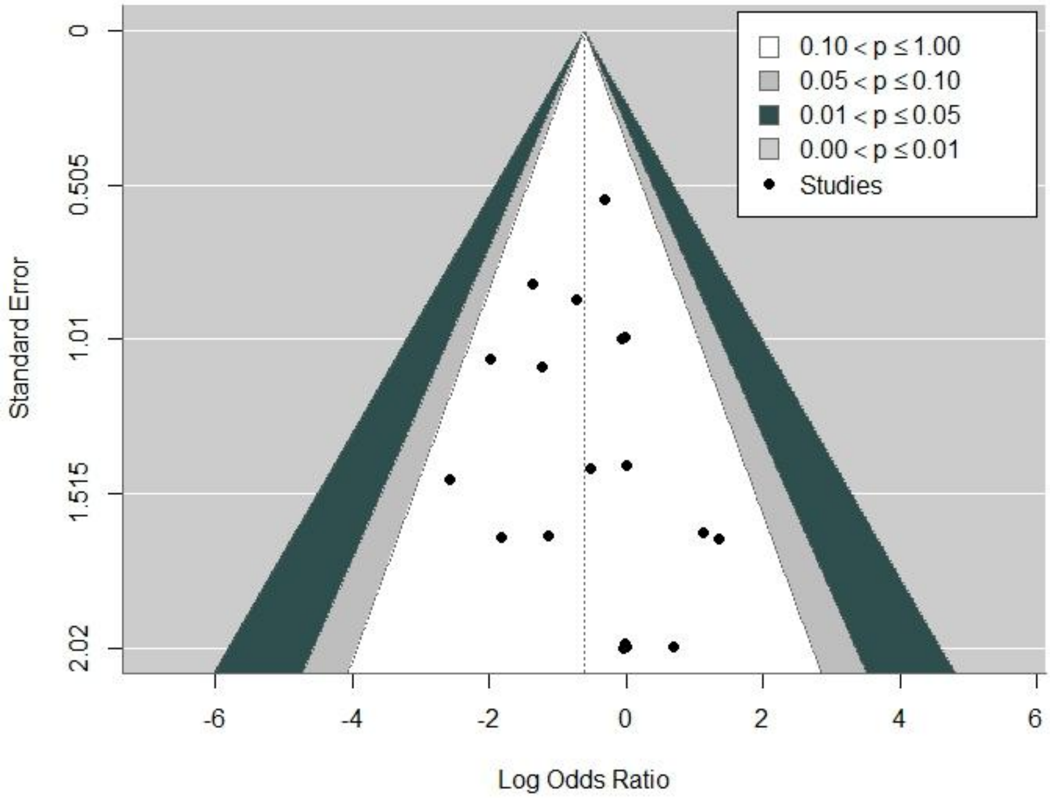

Supplementary Figure 11. Funnel plot for postoperative cerebrovascular events. The funnel plot is symmetric. One study (Camboni et al. 2009) had a relatively large weight compared to the rest of the studies (i.e., weight ≥ 3/k, so a weight at least 3 times as large as having equal weights across studies) but a sensitivity analysis excluding it was not deemed necessary since the examination of the studentized residuals revealed that none of the studies had a value larger than ±3.0781and hence there was no indication of outliers in the context of this model.

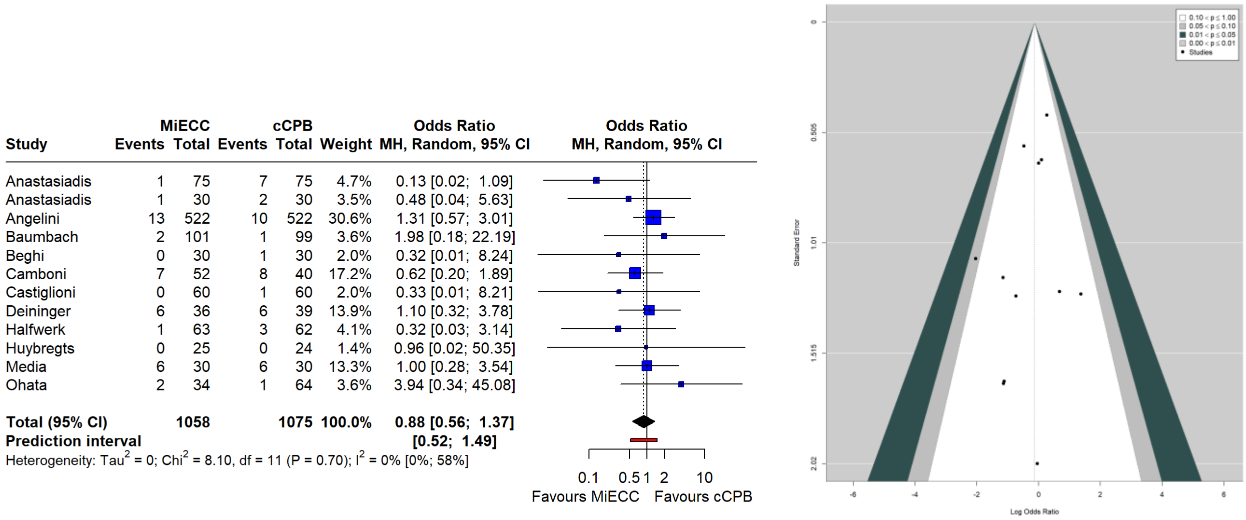

Supplementary Figure 12. (Left image): Forest plot of randomized trials comparing incidence of postoperative acute kidney injury in patients operated with minimal invasive extracorporeal circulation (MiECC) versus conventional cardiopulmonary bypass (cCPB). No difference is observed between groups (Right image). The funnel plot was symmetric.

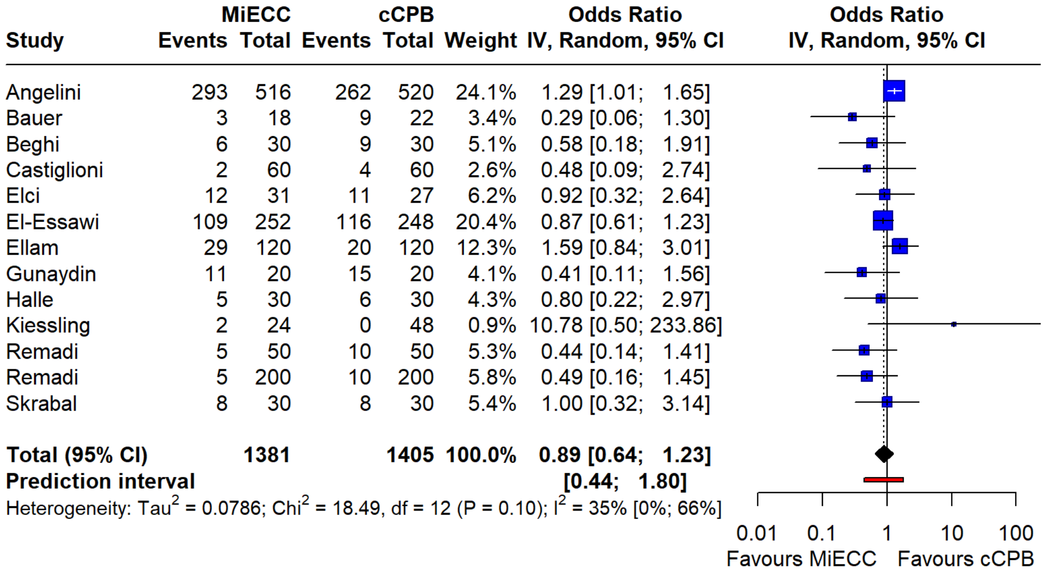

Supplementary Figure 13. Forest plot of randomized trials comparing need for inotropic support in patients operated with minimal invasive extracorporeal circulation (MiECC) versus conventional cardiopulmonary bypass (cCPB). No difference is observed between groups.

Study	MiECC Events	MiECC Total	cCPB Events	cCPB Total	Weight	Odds Ratio IV, Random, 95% CI
Angelini	1	515	2	515	17.5%	0.50 [0.05; 5.52]
Beghi	0	30	0	30	7.3%	1.00 [0.02; 52.04]
Halle	2	30	2	30	22.9%	1.00 [0.13; 7.60]
Kiessling	2	24	0	48	11.5%	10.78 [0.50; 233.86]
Remadi	1	50	3	50	18.8%	0.32 [0.03; 3.18]
Remadi	1	200	8	200	21.9%	0.12 [0.01; 0.97]
**Total (95% CI)**		**849**		**873**	**100.0%**	**0.59 [0.13; 2.63]**
**Prediction interval**						**[0.06; 5.67]**

Heterogeneity: $Tau^2 = 0.3398$; $Chi^2 = 6.26$, df = 5 (P = 0.28); $I^2 = 20\%$ [0%; 65%]

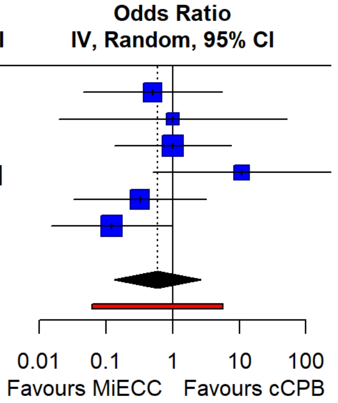

Supplementary Figure 14. Forest plot of randomized trials comparing incidence of low cardiac output syndrome in patients operated with minimal invasive extracorporeal circulation (MiECC) versus conventional cardiopulmonary bypass (cCPB). No difference is observed between groups.

Study	MiECC Events	MiECC Total	cCPB Events	cCPB Total	Weight	Odds Ratio MH, Random, 95% CI
Angelini	14	517	9	521	65.5%	1.58 [0.68; 3.69]
Beghi	0	30	0	30	3.0%	1.00 [0.02; 52.04]
Castiglioni	0	60	0	60	3.0%	1.00 [0.02; 51.22]
Gunaydin	2	20	5	20	14.9%	0.33 [0.06; 1.97]
Remadi	0	50	0	50	3.0%	1.00 [0.02; 51.38]
Remadi	0	200	1	200	4.6%	0.33 [0.01; 8.19]
Sakwa	1	102	1	97	6.0%	0.95 [0.06; 15.41]
**Total (95% CI)**		**979**		**978**	**100.0%**	**1.09 [0.59; 1.99]**
**Prediction interval**						**[0.44; 2.67]**

Heterogeneity: $Tau^2 = 0$; $Chi^2 = 3.00$, df = 6 (P = 0.81); $I^2 = 0\%$ [0%; 71%]

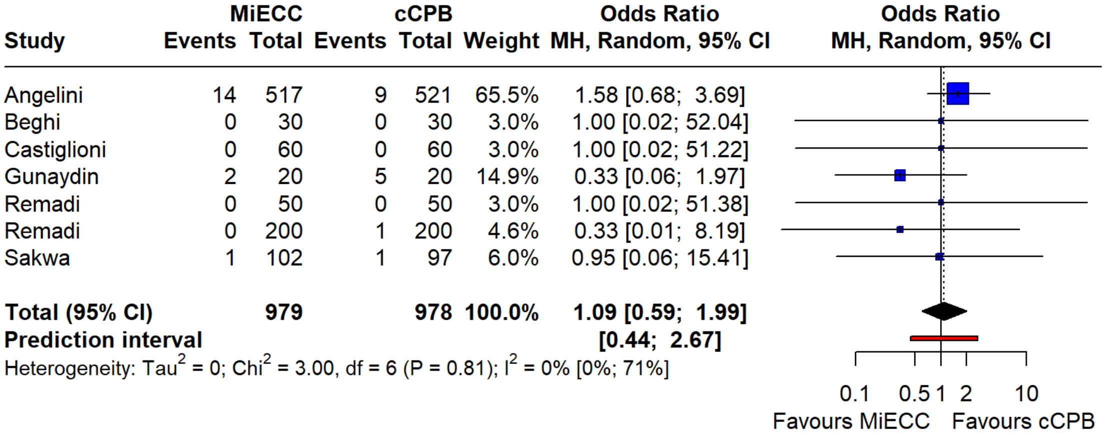

Supplementary Figure 15. Forest plot of randomized trials comparing incidence of intra-aortic balloon pump implantation in patients operated with minimal invasive extracorporeal circulation (MiECC) versus conventional cardiopulmonary bypass (cCPB). No difference is observed between groups.

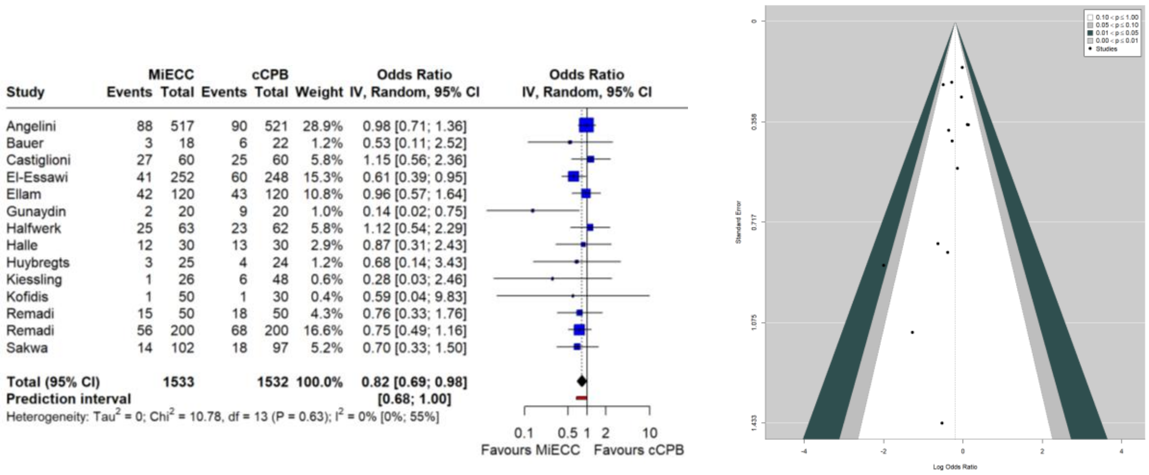

Supplementary Figure 16. (Left image): Forest plot of randomized trials comparing incidence of postoperative atrial fibrillation in patients operated with minimal invasive extracorporeal circulation (MiECC) versus conventional cardiopulmonary bypass (cCPB). A significant reduction (p=0.03) in the rate of postoperative atrial fibrillation is observed with the use of MiECC. (Right image): Funnel plot for postoperative atrial fibrillation. The funnel plot was asymmetric with an excess of negative effect small studies. One study (Angelini et al. 2024) had a relatively large weight compared to the rest of the studies and could be considered to be overly influential. A sensitivity analysis excluding this study did not result in any difference, both in direction and significance of the result.

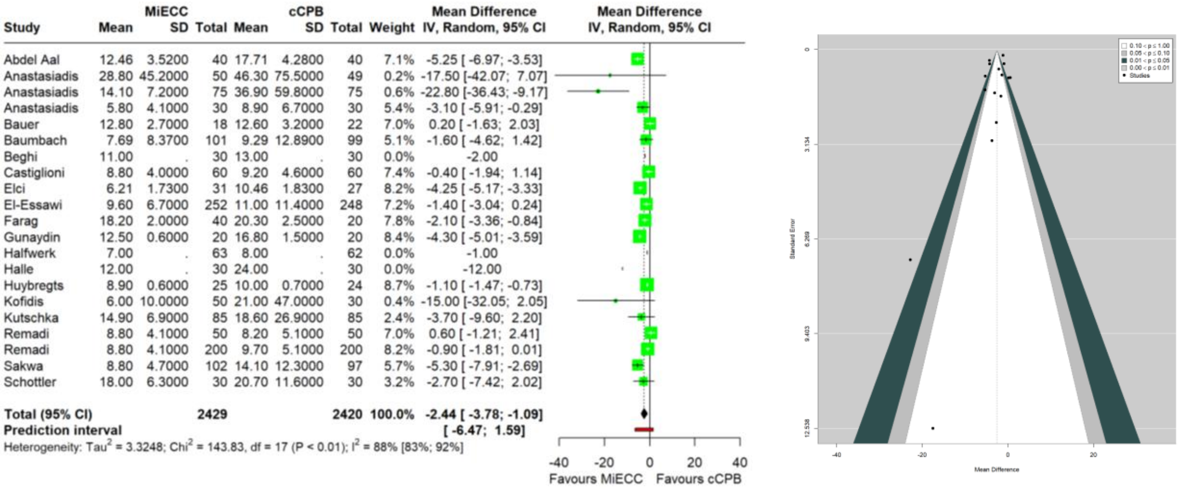

Supplementary Figure 17. (Left image): Forest plot of randomized trials comparing time on mechanical ventilation in patients operated with minimal invasive extracorporeal circulation (MiECC) versus conventional cardiopulmonary bypass (cCPB). MiECC was associated with significantly reduced need for mechanical ventilation (p=0.003). (Right image): The funnel plot was relatively symmetric with three small studies with excessively negative effect.

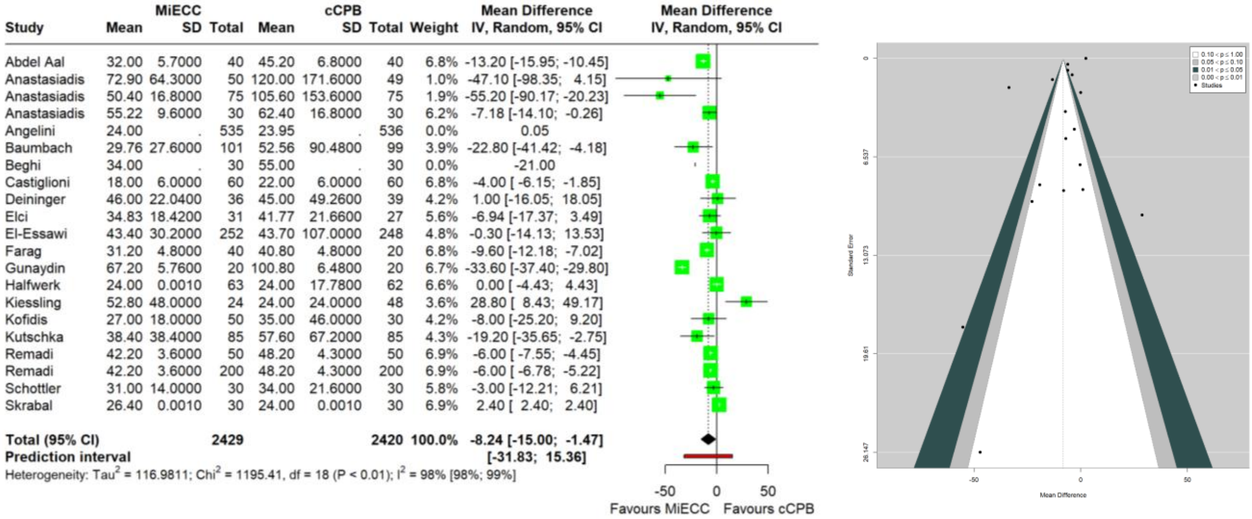

Supplementary Figure 18. (Left image): Forest plot of randomized trials comparing ICU stay in patients operated with minimal invasive extracorporeal circulation (MiECC) versus conventional cardiopulmonary bypass (cCPB). MiECC was associated with significantly reduced ICU stay (p=0.01). (Right image): The funnel plot was relatively symmetric with few studies laying out the contours but neither the rank correlation nor the regression test indicated any funnel plot asymmetry (p=0.7246 and p=0.9231, respectively).

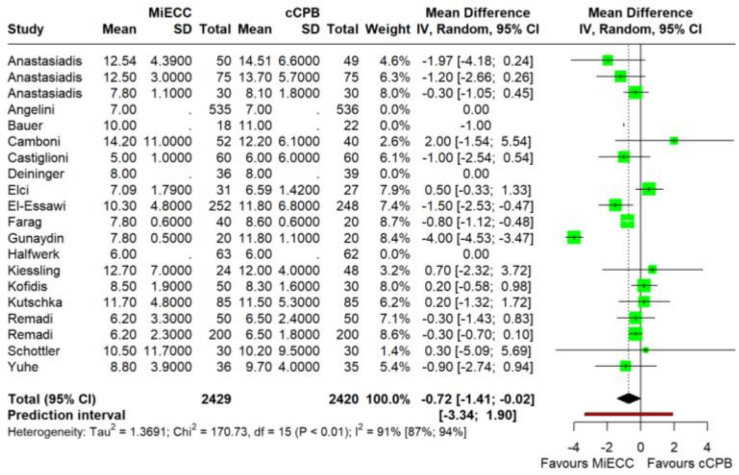

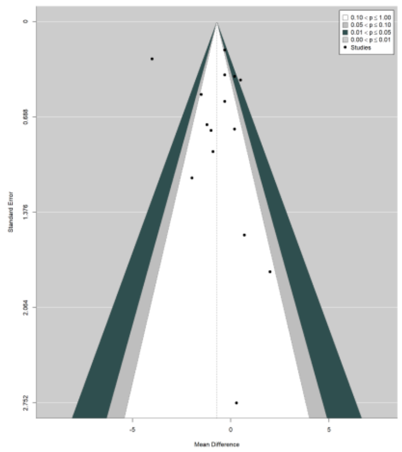

Supplementary Figure 19. (Left image): Forest plot of randomized trials comparing hospital stay in patients operated with minimal invasive extracorporeal circulation (MiECC) versus conventional cardiopulmonary bypass (cCPB). MiECC was associated with significantly reduced hospital stay (p=0.02). (Right image): The funnel plot was relatively symmetric with few studies laying out the contours.

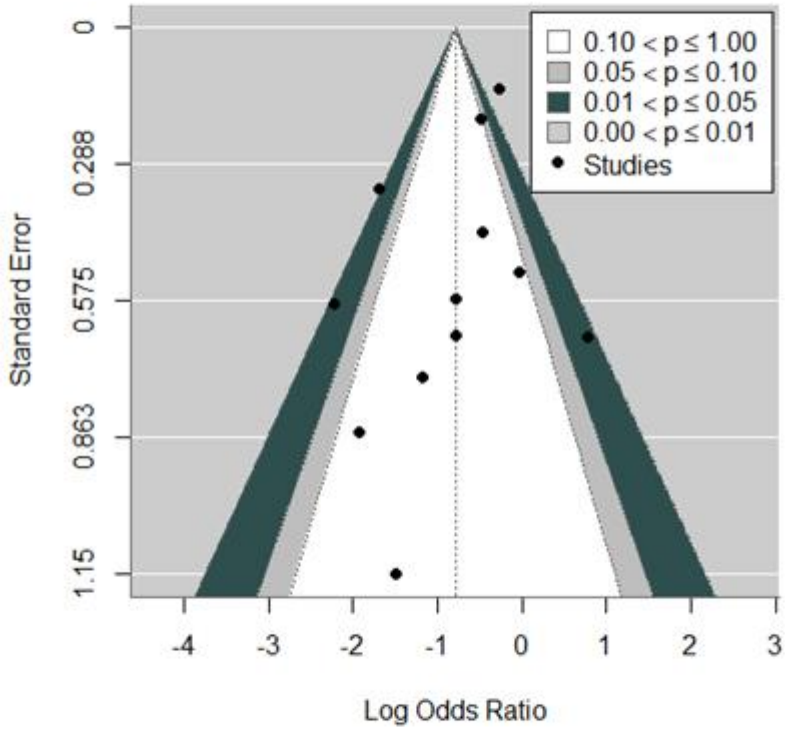

Supplementary Figure 20. Funnel plot for postoperative red blood cells (RBC) transfusion. The funnel plot was relatively symmetric. To explore the source of heterogeneity, a sensitivity analysis excluding studies indicated as highly influential from a Baujat plot was conducted (Gunaydin et al., Schottle et al. and El-Essawi et al.) and found a minimal amount of heterogeneity (I^2=5.8%, Q:7.8, df=8), with OR 0.65 (95% CI:0.51-0.83).

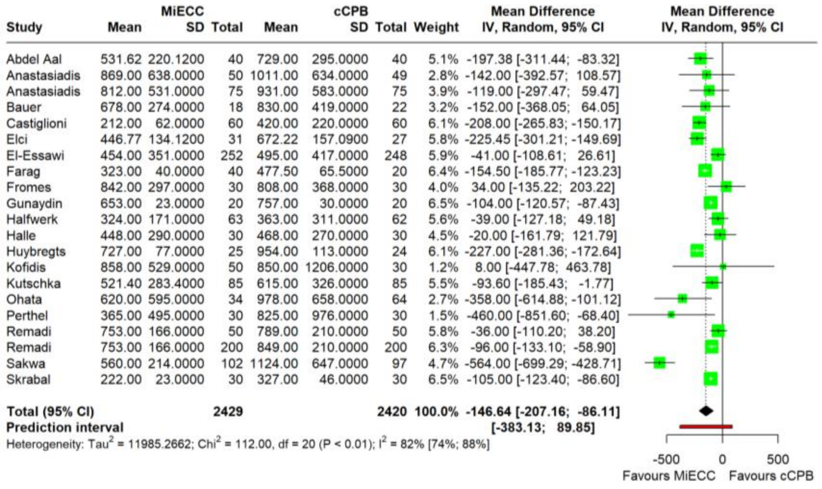

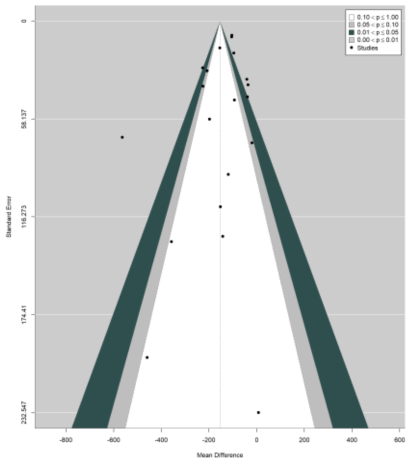

Supplementary Figure 21. (Left image): Forest plot of randomized trials comparing postoperative blood loss (ml) in patients operated with minimal invasive extracorporeal circulation (MiECC) versus conventional cardiopulmonary bypass (cCPB). A significant reduction ($p<0.001$) in the volume of postoperative blood loss is observed with the use of MiECC. (Right image): Funnel plot for postoperative blood loss (ml). The funnel plot was slightly asymmetric with missing small studies with positive effect but neither the rank correlation nor the regression test indicated any funnel plot asymmetry ($p=0.44$ and $p=0.4$, respectively).

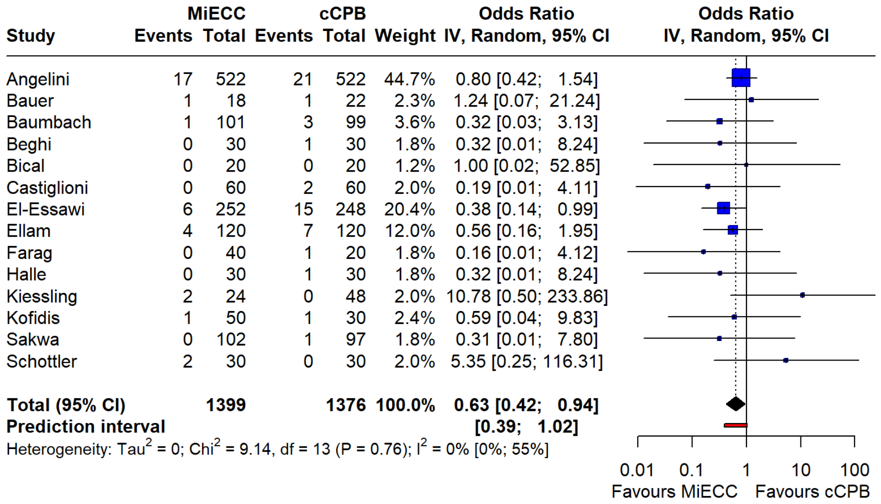

Supplementary Figure 22. Forest plot of randomized trials comparing incidence of re-exploration for bleeding in patients operated with minimal invasive extracorporeal circulation (MiECC) versus conventional cardiopulmonary bypass (cCPB). The rate of re-exploration is reduced with the use of MiECC, though not reaching statistical significance.

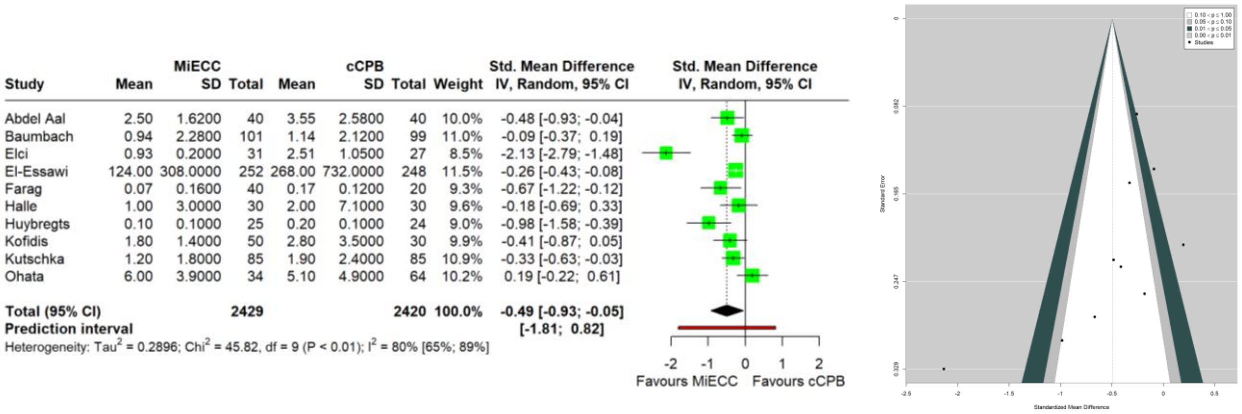

Study	MiECC Mean	MiECC SD	MiECC Total	cCPB Mean	cCPB SD	cCPB Total	Weight	Std. Mean Difference IV, Random, 95% CI
Abdel Aal	2.50	1.6200	40	3.55	2.5800	40	10.0%	-0.48 [-0.93; -0.04]
Baumbach	0.94	2.2800	101	1.14	2.1200	99	11.0%	-0.09 [-0.37; 0.19]
Elci	0.93	0.2000	31	2.51	1.0500	27	8.5%	-2.13 [-2.79; -1.48]
El-Essawi	124.00	308.0000	252	268.00	732.0000	248	11.5%	-0.26 [-0.43; -0.08]
Farag	0.07	0.1600	40	0.17	0.1200	20	9.3%	-0.67 [-1.22; -0.12]
Halle	1.00	3.0000	30	2.00	7.1000	30	9.6%	-0.18 [-0.69; 0.33]
Huybregts	0.10	0.1000	25	0.20	0.1000	24	9.0%	-0.98 [-1.58; -0.39]
Kofidis	1.80	1.4000	50	2.80	3.5000	30	9.9%	-0.41 [-0.87; 0.05]
Kutschka	1.20	1.8000	85	1.90	2.4000	85	10.9%	-0.33 [-0.63; -0.03]
Ohata	6.00	3.9000	34	5.10	4.9000	64	10.2%	0.19 [-0.22; 0.61]
**Total (95% CI)**			**2429**			**2420**	**100.0%**	**-0.49 [-0.93; -0.05]**
**Prediction interval**								**[-1.81; 0.82]**

Heterogeneity: $Tau^2 = 0.2896$; $Chi^2 = 45.82$, df = 9 (P < 0.01); $I^2 = 80\%$ [65%; 89%]

Supplementary Figure 23. (Left image): Forest plot of randomized trials comparing incidence of fresh frozen plasma (FFP) transfusion in patients operated with minimal invasive extracorporeal circulation (MiECC) versus conventional cardiopulmonary bypass (cCPB). A significant reduction ($p<0.001$) in the need for FFP transfusion is observed with the use of MiECC. (Right image): Funnel plot for FFP transfusion. The funnel plot was relatively symmetric. One study (Elci et al. 2019) was detected as potential outlier and a sensitivity analysis was conducted. Heterogeneity was explained in this model (I^2=0.005%, Q = 10.2, df = 7, p=0.1742).

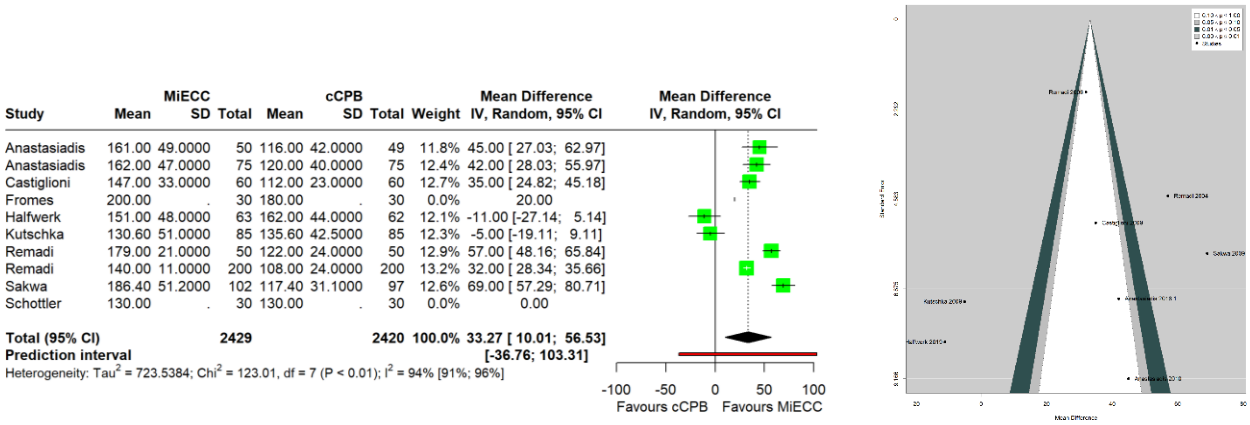

Study	MiECC Mean	MiECC SD	MiECC Total	cCPB Mean	cCPB SD	cCPB Total	Weight	Mean Difference IV, Random, 95% CI
Anastasiadis	161.00	49.0000	50	116.00	42.0000	49	11.8%	45.00 [27.03; 62.97]
Anastasiadis	162.00	47.0000	75	120.00	40.0000	75	12.4%	42.00 [28.03; 55.97]
Castiglioni	147.00	33.0000	60	112.00	23.0000	60	12.7%	35.00 [24.82; 45.18]
Fromes	200.00	.	30	180.00	.	30	0.0%	20.00
Halfwerk	151.00	48.0000	63	162.00	44.0000	62	12.1%	-11.00 [-27.14; 5.14]
Kutschka	130.60	51.0000	85	135.60	42.5000	85	12.3%	-5.00 [-19.11; 9.11]
Remadi	179.00	21.0000	50	122.00	24.0000	50	12.9%	57.00 [48.16; 65.84]
Remadi	140.00	11.0000	200	108.00	24.0000	200	13.2%	32.00 [28.34; 35.66]
Sakwa	186.40	51.2000	102	117.40	31.1000	97	12.6%	69.00 [57.29; 80.71]
Schottler	130.00	.	30	130.00	.	30	0.0%	0.00
**Total (95% CI)**			**2429**			**2420**	**100.0%**	**33.27 [10.01; 56.53]**
**Prediction interval**								**[-36.76; 103.31]**

Heterogeneity: $Tau^2 = 723.5384$; $Chi^2 = 123.01$, df = 7 (P < 0.01); $I^2 = 94\%$ [91%; 96%]

Supplementary Figure 24. (Left image): Forest plot of randomized trials comparing postoperative platelet count in patients operated with minimal invasive extracorporeal circulation (MiECC) versus conventional cardiopulmonary bypass (cCPB). A significantly increased postoperative platelet count is observed with the use of MiECC (p=0.01). (Right image): Funnel plot for postoperative platelet count. The funnel plot was relatively symmetric, though four studies laid outside the contours (Remadi et al. 2004, Kutschka et al. 2009, Sakwa et al. 2009 and Halfwerk et al. 2019).

Study	MiECC Mean	SD	Total	cCPB Mean	SD	Total	Weight	Mean Difference IV, Random, 95% CI
Abdel Aal	-7.81	3.0400	40	-12.71	2.9100	40	12.4%	4.90 [3.60; 6.20]
Angelini	-11.05	3.6900	535	-11.34	3.7900	536	13.1%	0.29 [-0.16; 0.74]
Castiglioni	3.00	4.4300	60	6.00	3.8300	60	12.1%	-3.00 [-4.48; -1.52]
Elci	-4.93	3.3000	31	-8.29	2.6900	27	12.0%	3.36 [1.82; 4.90]
Halfwerk	-5.00	2.5300	63	-8.00	2.5300	62	12.8%	3.00 [2.11; 3.89]
Huybregts	2.00	.	25	1.00	.	24	0.0%	1.00
Kiessling	-8.50	.	24	-7.20	.	48	0.0%	-1.30
Kutschka	-9.90	3.5400	85	-10.40	3.1800	85	12.7%	0.50 [-0.51; 1.51]
Sakwa	-2.60	2.8800	102	-6.10	3.0100	97	12.9%	3.50 [2.68; 4.32]
Yuhe	-10.40	3.1000	36	-14.70	3.6800	35	12.0%	4.30 [2.72; 5.88]
**Total (95% CI)**			**2429**			**2420**	**100.0%**	**2.10 [-0.09; 4.29]**
**Prediction interval**								**[-4.48; 8.68]**

Heterogeneity: Tau^2 = 6.3860; Chi^2 = 144.74, df = 7 (P < 0.01); I^2 = 95% [92%; 97%]

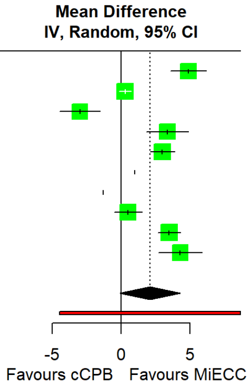

Supplementary Figure 25. Forest plot of randomized trials comparing hemodilution as detected with a drop in hematocrit in patients operated with minimal invasive extracorporeal circulation (MiECC) versus conventional cardiopulmonary bypass (cCPB). MiECC was associated with reduced hemodilution (p=0.058).

Study	MiECC Mean	SD	Total	cCPB Mean	SD	Total	Weight	Mean Difference IV, Random, 95% CI
Bical	116.00	46.0000	20	265.00	120.0000	20	19.5%	-149.00 [-205.32; -92.68]
Fromes	72.00	48.0000	30	220.00	103.0000	30	33.0%	-148.00 [-188.66; -107.34]
Kiessling	420.26	.	24	407.45	.	48	0.0%	12.81
Ohata	127.00	58.0000	34	240.00	100.0000	64	47.5%	-113.00 [-144.31; -81.69]
**Total (95% CI)**			**2429**			**2420**	**100.0%**	**-131.57 [-185.32; -77.82]**
**Prediction interval**								**[-363.24; 100.09]**

Heterogeneity: Tau^2 = 143.2211; Chi^2 = 2.32, df = 2 (P = 0.31); I^2 = 14% [0%; 91%]

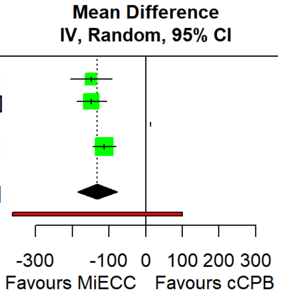

Supplementary Figure 26. Forest plot of randomized trials comparing polymorhonuclear elastase (PMNE) levels in patients operated with minimal invasive extracorporeal circulation (MiECC) versus conventional cardiopulmonary bypass (cCPB). MiECC was associated with significantly reduced levels of postoperative PMNE (p=0.008).

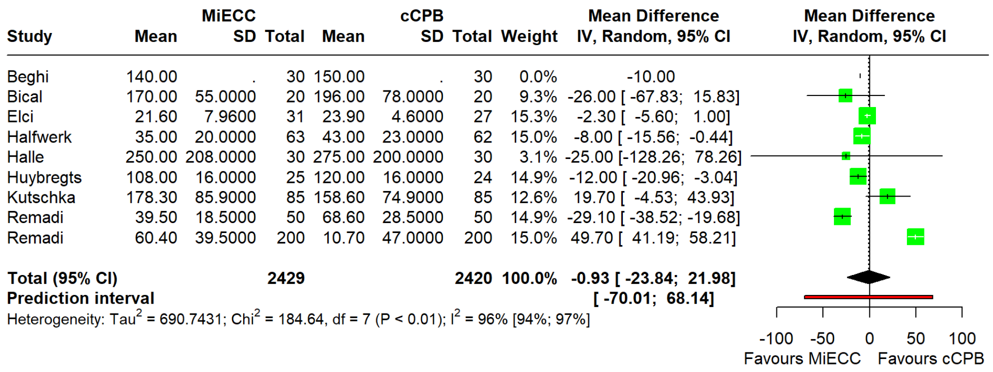

Supplementary Figure 27. Forest plot of randomized trials comparing levels of c-reactive protein (CRP) in patients operated with minimal invasive extracorporeal circulation (MiECC) versus conventional cardiopulmonary bypass (cCPB). No difference is observed between groups.

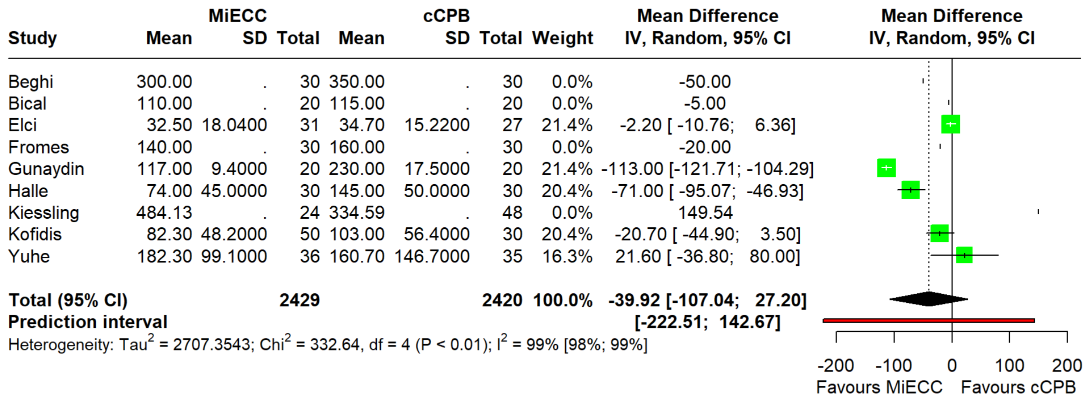

Supplementary Figure 28. Forest plot of randomized trials comparing levels of interleukin-6 (IL-6) in patients operated with minimal invasive extracorporeal circulation (MiECC) versus conventional cardiopulmonary bypass (cCPB). No difference is observed between groups.
